# Whole genome sequencing and analysis reveal insights into the genetic structure, diversity and evolutionary relatedness of *luxI* and *luxR* homologs in bacteria belonging to the *Sphingomonadaceae* family

**DOI:** 10.3389/fcimb.2014.00188

**Published:** 2015-01-08

**Authors:** Han Ming Gan, Huan You Gan, Nurul H. Ahmad, Nazrin A. Aziz, André O. Hudson, Michael A. Savka

**Affiliations:** ^1^School of Science, Monash University MalaysiaPetaling Jaya, Malaysia; ^2^Genomics Facility, Monash University MalaysiaPetaling Jaya, Malaysia; ^3^Thomas H. Gosnell School of Life Sciences, Rochester Institute of Technology RochesterNY, USA

**Keywords:** *luxI/R*, *luxR* solos, *Novosphingobium*, quorum-sensing, *Sphingomonadaceae*, phylogenetic, whole genome sequencing

## Abstract

Here we report the draft genomes and annotation of four *N*-acyl homoserine lactone (AHL)-producing members from the family *Sphingomonadaceae*. Comparative genomic analyses of 62 *Sphingomonadaceae* genomes were performed to gain insights into the distribution of the canonical *luxI/R*-type quorum sensing (QS) network within this family. Forty genomes contained at least one *luxR* homolog while the genome of *Sphingobium yanoikuyae* B1 contained seven Open Reading Frames (ORFs) that have significant homology to that of *luxR*. Thirty-three genomes contained at least one *luxI* homolog while the genomes of *Sphingobium* sp. SYK6, *Sphingobium japonicum*, and *Sphingobium lactosutens* contained four *luxI*. Using phylogenetic analysis, the sphingomonad LuxR homologs formed five distinct clades with two minor clades located near the plant associated bacteria (PAB) LuxR solo clade. This work for the first time shows that 13 *Sphingobium* and one *Sphingomonas* genome(s) contain three convergently oriented genes composed of two tandem *luxR* genes proximal to one *luxI* (*luxR-luxR-luxI*). Interestingly, *luxI* solos were identified in two *Sphingobium* species and may represent species that contribute to AHL-based QS system by contributing AHL molecules but are unable to perceive AHLs as signals. This work provides the most comprehensive description of the *luxI/R* circuitry and genome-based taxonomical description of the available sphingomonad genomes to date indicating that the presence of *luxR* solos and *luxI* solos are not an uncommon feature in members of the *Sphingomonadaceae* family.

## Introduction

Members of the *Sphingomonadaceae* family are Gram-negative glycosphingolipid-containing bacteria that belong to the α-4 subclass of proteobacteria (Yabuuchi et al., 1990). This family possesses a variety of metabolic capabilities that are potentially advantageous pertaining to a variety of bioremediation capabilities (White et al., 1996). Based on phylogenetic, chemotaxonomic and phenotypic observations, the *Sphingomonas* genus has been expanded to include three new genera, *Sphingobium, Novosphingobium* and *Sphingopyxis* (Yabuuchi et al., 1990). Recently, a fifth genus was added to include, *Sphingosinicella* (Maruyama et al., 2006; Geueke et al., 2007; Yoon et al., 2008; Yasir et al., 2010).

Regarding niche, sphingomonads have been isolated from a variety of terrestrial and aquatic environments, including; water supplies, respirators, blood, wounds, dialysis equipment, patients with septicemia, peritonitis, meningitis, and wound infections, soils, deep subsurface sediments, corroding copper pipes and in plants (White et al., 1996; Gan et al., 2009).

Members of the *Sphingomonas* genus are able to catabolize a wide range of natural recalcitrant and anthropogenic compounds including; biphenyl, naphthalenes, pyrene, furans, oestradiol, polyethylenglycols, chlorinated phenols, and various biocides such as carbofuran, 2,4-D and mecoprop (Ogramab et al., 2000; Basta et al., 2004; Stolz, 2009). It was shown that the biphenyl- and naphthalene-degrading *Sphingomonas aromaticivorans* F199 strain and other sphingomonads that degrade additional xenobiotic compounds contain large plasmids encoding the catabolic pathways (Romine et al., 1999; Ogramab et al., 2000; Basta et al., 2004, 2005). Evidence also supports that these replicons can only be transferred among sphingomonads (Ogramab et al., 2000; Basta et al., 2004) by conjugal transfer and that gene and gene cluster rearrangements in the plasmids occur post conjugation (Tiirola et al., 2002). The presence of multiple insertion elements in sphingomonads suggests a role in the establishment of degradative pathways and in plasmid rearrangements and differences in gene cluster localization in members of the *Sphingomonadaceae* family (Dogra et al., 2004; Muller et al., 2004; Thiel et al., 2005). A recent study comparing the genomes of 26 sphingomonads suggests diverse adaptations and biodegradative capabilities in this group within the phylum *Alphaproteobacteria* (Aylward et al., 2013). Given this complexity in niche environments, biodegradation capabilities and genome rearrangements, the whole genome sequencing of additional sphingomonads has the potential to enhance our understanding of the diversity within this group and may contribute to important biotechnological applications such as bioremediation in the future.

Quorum sensing (QS) is a system commonly employed by bacteria to monitor its cell density prior to regulating gene expression (Fuqua et al., 1994; Miller and Bassler, 2001; Waters and Bassler, 2005; Schuster et al., 2013). In one type of QS system from Gram-negative bacteria, the bacteria produce and detect chemical signals called *N*-acyl-homoserine lactones (AHL). These signals are produced by the enzyme AHL synthase, a member of the LuxI-type protein family. The AHL compounds are detected by a transcriptional regulator belonging to the LuxR-type family. A typical AHL-QS system contains a LuxI and a LuxR protein that are usually in a genomic context regarding proximity of the genes on the chromosome (Choudhary et al., 2013). Upon reaching a concentration threshold measured by the cell density, the AHL signal is detected by the cognate LuxR and can activate population-wide-responses leading to the coordination of gene activation or repression. In Gram-negative bacteria, AHL dependent QS regulation is used to regulate the production of diverse responses such as; the activation of virulence factors, conjugation, the production of antimicrobial metabolites, the regulation of enzyme secretion, the production of bioluminescence and the anabolism of polysaccharide production which is correlated to biofilm formation (Miller and Bassler, 2001; Fuqua and Greenberg, 2002; Waters and Bassler, 2005).

Besides the presence of the canonical *luxI/luxR* pairs, many bacteria contain additional *luxR* transcriptional regulators that are not in a genomic context regarding proximity to a *luxI* gene. These unpaired *luxR* genes have been termed solos and orphans and are homologous to QS LuxR-type transcriptional regulators in that LuxR solos contain the AHL-binding domain at the N terminus and a DNA-binding helix-turn-helix (HTH) domain at the C terminus (Fuqua, 2006; Case et al., 2008; Subramoni and Venturi, 2009; Tsai and Winans, 2010; Cude and Buchan, 2013; Gonzalez and Venturi, 2013). The solo LuxR-type transcriptional activators increase the regulatory range by responding to endogenously produced AHLs and by “listening-in” on exogenous signals produced by other bacteria. Recently, a subfamily of LuxR solos have been found that respond to plant-produced compounds and were subsequently named the plant associated bacteria (PAB) luxR solos (Ferluga et al., 2007; Zhang et al., 2007). In addition, LuxI solos were identified first by Zan et al. (2012) and have also been subsequently identified in *Sulfitobacter, Ruesgeria*, and *Phaeobacter* genera all within the *Roseobacter* clade (Cude and Buchan, 2013).

Members of the sphingomonads have been shown to synthesize AHL signals (D'angelo-Picard et al., 2005; Gan et al., 2009; Huang et al., 2013; Schaefer et al., 2013). Previous work by our group have, isolated, identified, sequenced and annotated the genome of an AHL-producing *Novosphingobium* sp. Rr 2-17 isolated from a grapevine tumor (Gan et al., 2009, 2012). Comparative genomic analysis of Rr 2-17 and five additional members from the genus *Novosphingobium* validated the presence of canonical *luxI/luxR* pairs. Furthermore, a putative *luxR* solo in strain PP1Y of the *Novosphingobium* genus was identified (Gan et al., 2013). Our initial and continuing work with a group of sphingomonads documented to degrade natural and anthropogenic compounds identified a subset of four sphingomonads capable of producing AHL QS signals. We decided to sequence their whole genomes to corroborate AHL-producing phenotype with the presence of *luxI* and *luxR* homologs in the whole genomes and more importantly to contribute molecular resources for future genetic work pertaining to microbial-based bioremediation.

Leveraging on the expansion of microbial genomics data, the additional objectives of this study are to (1) provide an updated genomic distribution of *luxI/R* homologs in the *Sphingomonadaceae* family, (2) update and validate sphingomonad taxonomy using genome-based approach, (3) provide a comprehensive LuxR phylogeny and (4) identify putative LuxR solos and LuxI solos in the currently sequenced sphingomonads.

## Materials and methods

### Strains, culture conditions and extract preparation

The bacterial strains (kindly provided by Andreas Stolz, Institut fur Mikrobiologie, Universitat Stuttgart, Stuttgart, Germany) used in this work were cultured on R2A minimal agar media. To prepare extracts for the detection of AHL compounds, the four sphingomonads were grown on potato dextrose agar medium for 4 days and were resuspended in 10 mls sterile purified water. Equal volume of acidified ethyl acetate (aEtOAc) was added to the resuspended bacteria and the mixture was agitated for 3 h at 25°C with shaking at 150 rpm followed by centrifugation to separate the aqueous phase from the aEtOAc phase. Under these conditions, AHLs partition into the non-polar aEtOAC phase. The aEtOAc was aspirated off, dried in a Savant speed-vac and resuspended in aEtOAc to produce a 20-fold concentrated aEtOAc extracts. These extracts were used in AHL detection assays.

*E. coli* JM109, *Agrobacterium tumefaciens* A136 and *Chromobacterium violaceum* CV026 were grown in Luria-Bertani (LB). Each bacterial biosensor reporter strain used in this work is listed in Supplemental Table 1 along with its AHL receptor protein and cognate AHL signal. All media and growth conditions are as previously described by our group (Scott et al., 2006; Gan et al., 2009; Lowe et al., 2009; Savka et al., 2011).

### Bioassays for AHL QS signal detection using AHL-dependent biosensor strains

An overnight culture of these four biosensors were grown in LB with the appropriate antibiotic and diluted 1:10 in LB and 200 μl of the diluted cell suspension was added to the round bottom tubes (12 × 50 mm) containing dried aEtOAc samples or pure AHL signals as controls. Cognate AHL signal for *E. coli* biosensors JM109 (pSB401) was 3-oxo-C6-HSL at 50 nM; for JM109 (pSB536) was C4-HSL at 1 μM; for JM109 (pSB1075) was 3-oxo-C12-HSL at 1 nM, unless otherwise noted. For the *A. tumefaciens* A136 biosensor pure C8-HSL was used at 50 nM. Tubes were incubated at 30°C with shaking for 5 to 6 h before bioluminescence was measured using a Turner Designs TD 20/20 luminometer. The TD 20/20 luminometer was adjusted to different sensitivities due to the varying responses of the JM109 series of biosensors to their cognate AHL signal. Unless noted, relative light units (RLU) measurements were made at 30.0, 39.9, 50.1, and 30.0% sensitivity for LuxR-, AhyR, LasR, and TraR-based biosensors, respectively. Luminescence is measured and given in RLU per triplicate sample. RLUs were determined with a 20-s integration period. Mean values of the RLUs were obtained with three independent biological samples.

For “T”-streak assays, the *Chromobacterium violaceum* colorless mutant, CV026 was used. In the presence of exogenous QS signals CVO26 produces the purple pigment violacein, indicating the presence of AHL in the sample. *C. violaceum* wild type strain was used as a positive control. *E. coli* DH5α was the negative control in the T-streak plate assays. The biosensor, controls, and samples were grown on tryptone—yeast extract medium mixed with PDA medium (1:1, v/v). Each isolate was tested at least two times using the “T”-streak bioassay. The whole cell AHL-dependent biosensor assays were performed as previously described by our group (Scott et al., 2006; Gan et al., 2009; Lowe et al., 2009; Savka et al., 2011).

### Whole genome sequencing, assembly and annotation

Genomic DNA was extracted using the GenElute™ (Sigma-Aldrich, St. Louis, MO) and converted into next generation sequencing library using Nextera XT (Illumina, San Diego, CA) according to the manufacturer's instructions. Whole genome sequencing was performed using the MiSeq (Illumina, San Diego, CA) at the Monash University Malaysia Genomics Facility. The raw data for each bacterium were error-corrected and assembled using Spades v2.5 (default setting) (Bankevich et al., 2012). The generated contigs were scaffolded and gap-closed using SSPACE and GAPFiller respectively (Boetzer et al., 2011; Boetzer and Pirovano, 2012). Genome annotation was performed using Prokka and InterProScan5 (Jones et al., 2014; Seemann, 2014).

### Whole genome-based phylogeny assignment

Publicly available complete and draft genome sequences (<250 contigs) from the genus *Novosphingobium, Sphingomonas, Sphingopyxis*, and *Sphingobium* were downloaded. Subsequently, gene/protein prediction was performed using Prodigal2.60 (default setting) (Hyatt et al., 2010). PhyloPhlAn was used to construct phylogenetic tree from the resulting predicted proteins based on 400 highly conserved microbial proteins (Segata et al., 2013).

### Systematic bioinformatics identification of LuxI, LuxR and LuxR solo homologs

**Figure 1:**
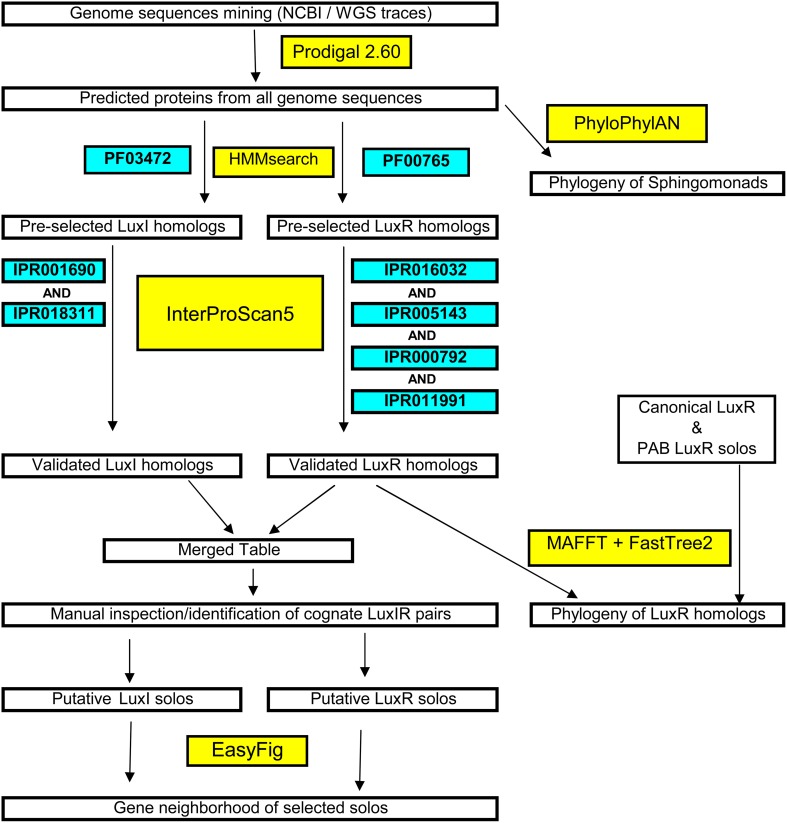
**Flowchart of the systematic and stringent bioinformatics methodology used in this study for the identification of *luxI/R* in sphingomonads and large-scale phylogenetic/phylogenomic tree construction**.

A systematic methodology for the accurate and stringent identification of LuxI, LuxR, and LuxR solo homologs is presented in Figure 1. Briefly, the predicted proteomes were scanned for protein family domain (PFAM) specifically the autoinducer synthase domain (PFAM signature: PF00765) and the autoinducer binding domain (PFAM signature: PF03472) that are universally present in reported LuxR and LuxI homologs, respectively, using profile hidden Markov models-based similarity search (*E*-value <1e-5). The short listed candidates were further annotated using the more time consuming but comprehensive InterProScan5. To qualify as an authentic LuxR homolog, the shortlisted protein must contain four signature LuxR homolog Interproscan identifiers e.g., IPR005143 (autoinducer binding), IPR016032 (Signal transduction response regulator, C-terminal effector), IPR011991 (Winged helix-turn-helix DNA-binding domain), and IPR000792 (Transcription regulator LuxR, C-terminal) that are universally present in functionally validated LuxR homologs. An authentic LuxI homolog on the other hand, must contain both IPR001690 (Autoinducer synthesis protein) and IPR018311 (Autoinducer synthesis, conserved site). Cognate LuxI and LuxR homologs were then manually identified based on the coordinate and close proximity of their respective protein-coding genes.

### Maximum likelihood approximation of the LuxR phylogeny

Functionally validated LuxR homologs, PAB LuxR solos and the putative sphingomonad LuxR homologs were combined and aligned with MAFFT-LINSI using the default setting (Katoh and Standley, 2013). The resulting protein alignment was then used as the input for maximum likelihood phylogenetic analysis using FastTree2 (Price et al., 2010). The constructed tree was visualized and graphically edited using FigTree (Rambaut, 2014).

### Visualization of *LuxR* and *LuxI* solos gene neighborhood, LuxR homologs alignment and pairwise identity matrix construction

Contigs containing the identified *luxR* solo genes were extracted from the genome, annotated with Prokka (default setting) and subsequently visualized in EasyFig (Sullivan et al., 2011). Additionally, sphingomonad LuxR homologs clustered with the PAB LuxR solos were aligned with MAFFT-LINSI (Katoh and Standley, 2013) and visualized using ALINE (Bond and Schuttelkopf, 2009). Pairwise identity matrix for selected LuxR homologs was constructed using SDT (Muhire et al., 2014).

## Results

### Genome statistics of the four newly sequenced sphingomonads and their ability to produce AHL signals

The genome assembly and annotation statistics of four genomes of *Sphingomonas* known for their biodegradation ability in addition to their isolation source and notable features are presented in Table 1. Culture extracts prepared from each of the four sphingomonads strains in this study chosen for whole genome sequencing activated at least two AHL-dependent whole cell bacterial biosensors (Supplemental Table 1). *Sphingomonas paucimobilis* EPA505 activated light production in the TraR-based *Agrobacterium* A136 and in the LasR-based *E. coli* JM109 (pSB1075) biosensors and activated pigment synthesis in the CviR-based *Chromobacterium* biosensor. The *Sphingobium herbicidovorans* NBRC16415, *Sphingobium yanoikuyae* B1 and *Novosphingobium resinovorum* KF1 activated light production in the TraR- and activated pigment synthesis in the CviR-based biosensors (Table 2). These results are consistent with findings by others that AHL QS signal production in members of the sphingomonad group is not uncommon (D'angelo-Picard et al., 2005; Gan et al., 2009; Huang et al., 2013; Schaefer et al., 2013).

**Table 1:** **Strain information, genome assembly and annotation statistics of the sequenced sphingomonads used in this study**.

**Strain**	A. N.^*^	**Size (bp)**	**No. of contigs**	**N50**	**GC%**	**Some compounds degraded**	**Isolation source**	**References**
*Novosphingobium resinovorum* KF1	JFYZ01	6,304,486	115	171,782	65.06	2, 3, 4,6-tetrachlorophenol	Fluidized-bed reactor	Takeuchi et al., 2001
*Sphingobium herbicidovorans* NBRC16415	JFZA01	4,032,326	62	178,990	62.44	2, 4-dichlorophenoxyacetate	Soil	Zipper et al., 1996
*Sphingobium yanoikuyae* B1	JGVR01	5,683,787	116	158,314	63.94	Toluene, biphenyl	Polluted stream	Yabuuchi et al., 1990
*Sphingomonas paucimobilis* EPA505	JFYY01	4,874,185	81	285,203	63.93	Fluoranthene, naphthalene(s)	Creosote waste site	Muller and Wittmann-Liebold, 1997

* *A.N., Accession Number*.

**Table 2:** Production of *N*-acyl-homoserine lactones by four newly sequenced strains of the *Sphingomonas, Sphingobium* and *Novosphingobium* group assayed by five AHL-dependent biosensor strains^*^.

**Genus and species/strain**	**AhyR^‡^**	**LuxR^‡^**	**TraR^‡^**	**LasR^‡^**	**CviR^#^**
*Sphingomonas paucimobilis* EPA505	−	−	+++	+	+++
*Sphingobium herbicidovorans* NBRC16415	−	−	++	−	+++
*Sphingobium yanoikuyae* B1	−	−	++	−	+
*Novosphingobium resinovorum* KF1	−	−	+++	−	+

* *Abbreviations include: AhyR, AHL receptor from Aeromonas hydrophilia; LuxR, from Vibrio fisheri; TraR, from Agrobacterium tumefaciens; LasR, from Pseudomonas aeruginosa; CviR, from Chromobacterium violaceum*.

‡ *Scores for biosensor detection of AHL in strain extracts are based on the following criteria: -, < 2-fold higher than background levels of relative light units (RLU) bioluminescence; +, > 2-fold higher than background RLUs; ++, 50 to 75-fold higher than background RLUs; +++, > 75-fold higher than background in RLUs*.

# *CviR, AHL-dependent receptor of biosensor strain CV026. Scores were relative violacein pigment production in T-streak bioassays on PDA/TYE (1:1) agar media*.

### Phylogenomic analysis of currently sequenced sphingomonad

**Figure 2:**
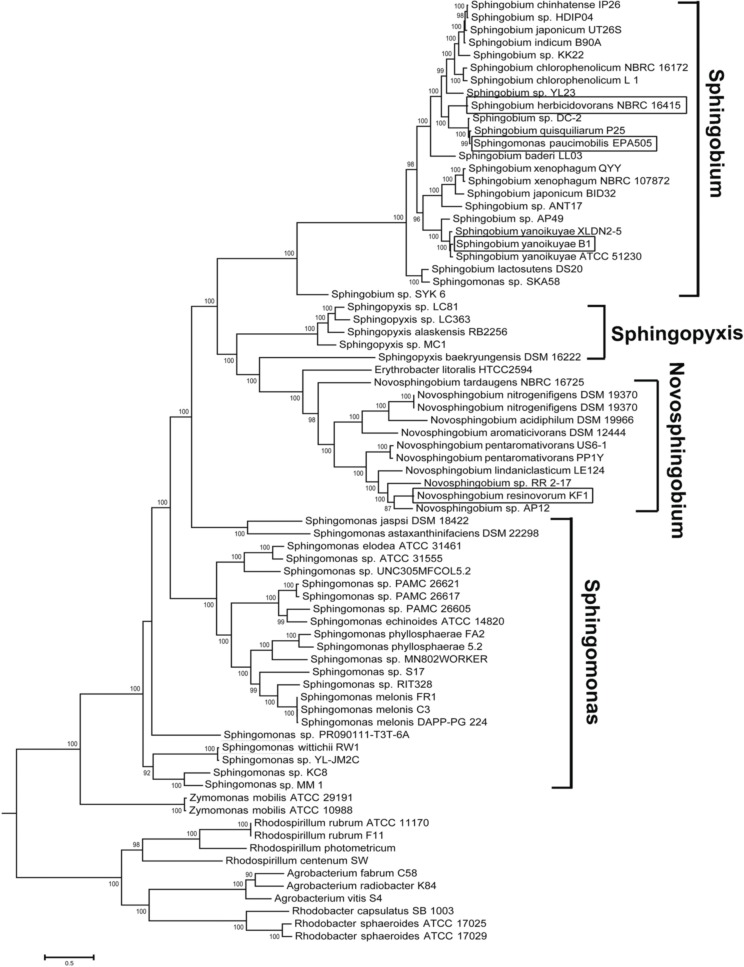
**Phylogenomic tree depicting the evolutionary relationship of currently sequenced sphingomonads based on approximately 400 conserved single-copy genes**. The four whole genomes sequenced in study were shown in rectangle boxes. Selected members from the genera *Rhodospirillum, Agrobacterium and Rhodobacter* were designated as outgroup. Bootstrap support of less than 50% was not shown.

Analysis of the currently sequenced sphingomonads (Figure 2 and see Supplemental Table 4 for accession number) indicates that there is a sequencing bias toward the genera *Sphingobium, Novosphingobium*, and *Sphingomonas*. Very recently, our group sequenced and annotated the genomes of two additional cave *Sphingopyxis* genomes that enabled the expansion of the taxon sampling size (Gan et al., 2014). Species from the genera *Sphingobium* and *Novosphingobium* form robust monophyletic lineages with extremely high (>90%) nodal support. Based on phylogenomic analysis, the *Sphingobium* clade is the sister group to the clade of *Sphingopyxis* and *Novosphingobium*. Notably, species from the genus *Sphingomonas* display considerable paraphyletic distributions, indicating incongruence between molecular and biochemical-based taxonomic assignment. Phylogenomic analysis also suggests that *Sphingomonas* sp. SKA85 and the classic *Sphingomonas paucimobilis* EPA505 (Nohynek et al., 1996) may have been misclassified at the genus level as evidenced by its tight clustering within the *Sphingobium* group.

### The presence and composition of sphingomonad *LuxR* and *LuxI* homologs are diverse

The analysis of 62 sphingomonads genomes provides genetic evidence that QS is a common trait within the family. 40 of the 62 genomes analyzed contain at least one putative *luxI* or *luxR* homolog with 33 of them containing at least 1 putative canonical *luxI/R* homolog pair (Table 3 and See Supplemental Tables 2, 3 for a complete information of the identified *luxR* and *luxI* homologs). The non-universal presence of QS genes in members of the same species e.g., *Sphingobium yanoikuyae* and *Sphingobium xenophagum* may imply that QS is a trait that is subject to purifying selection. It is also worth noting that members of the currently sequenced *Sphingomonas* have a relatively incomplete *lux*-based QS capacity as evidenced by the sparse presence of *luxI* and *luxR* homologs in this genus.

**Table 3:** **Distribution and organization of the *luxI* and *luxR* homologs and the presence of direct double *luxR-luxI* topology identified in the whole genome sequences of 40 sphingomonads**.

**Organism**	**Canonical *luxR, luxI***	***luxR-luxR-luxI* and neighborhood variation^#^**	**Unpaired *luxI* (solos)**	**Unpaired *luxR* (solos)**
NOVOSPHINGOBIUM (7/11) ^‡^ [6,0,0,3] ^*^				
*Novosphingobium lindaniclasticum* LE124	1	0	0	0
*Novosphingobium pentaromativorans* PP1Y	2	0	0	1
*Novosphingobium pentaromativorans* US6-1	1	0	0	0
*Novosphingobium resinovorum* KF1**^†^**	2	0	0	2
*Novosphingobium* sp. AP12	1	0	0	0
*Novosphingobium* sp. RR 2-17	1	0	0	0
*Novosphingobium tardaugens* NBRC 16725	0	0	0	2
SPHINGOBIUM (18/24)^‡^ [11,11,8,12] ^*^				
*Sphingobium baderi* LL03	0	2 with T3 and T5	1	3
*Sphingobium chinhatense* IP26	1	0	1	0
*Sphingobium chlorophenolicum* NBRC 16172	2	0	1	1
*Sphingobium chlorophenolicum* L1	0	1 with T1	1	1
*Sphingobium herbicidovorans* NBRC 16415**^†^**	1	1 with T3	0	1
*Sphingobium indicum* B90A	0	1 with T1	1	1
*Sphingobium japonicum* UT26S	2	1 with T1	0	1
*Sphingobium lactosutens* DS20	2	1 with T1	0	0
*Sphingobium* sp. ANT17	1	0	0	0
*Sphingobium* sp. AP49	1	0	0	0
*Sphingobium* sp. DC-2	0	0	0	1
*Sphingobium* sp. HDIP04	0	1 with T4	1	1
*Sphingobium* sp. KK22	1	1 with T3	2	0
*Sphingobium* sp. SYK6	2	1 with T1	0	1
*Sphingobium* sp. YL23	0	1 with T3	0	0
*Sphingobium xenophagum* QYY	0	1 with T2	0	1
*Sphingobium yanoikuyae* ATCC 51230	2	0	1	5
*Sphingobium yanoikuyae* B1**^†^**	2	0	0	1
SPHINGOMONAS (11/22)^‡^ [5,2,0,7] ^*^				
*Sphingomonas elodea* ATCC 31461	0	0	0	1
*Sphingomonas paucimobilis* EPA505**^†^**	2	0	0	1
*Sphingomonas* sp. KC8	1	0	0	0
*Sphingomonas* sp. MM1	0	1 with T6	0	0
*Sphingomonas* sp. PAMC 26617	0	0	0	1
*Sphingomonas* sp. PAMC 26621	0	0	0	1
*Sphingomonas* sp. S17	0	0	0	1
*Sphingomonas* sp. SKA58	1	1 with T1	0	0
*Sphingomonas* sp. UNC305MFCOL5.2	1	0	0	0
*Sphingomonas* sp. YL-JM2C	2	0	0	2
*Sphingomonas wittichii* RW1	0	0	0	3
SPHINGOPYXIS (4/5)^‡^ [4,0,0,0] ^*^				
*Sphingopyxis alaskensis* RB2256	2	0	0	0
*Sphingopyxis* sp. LC363	1	0	0	0
*Sphingopyxis* sp. LC81	2	0	0	0
*Sphingopyxis* sp. MC1	1	0	0	0

‡ *(Number of genomes luxI and/or luxR)/(Total genome)*.

* *Number of genomes with luxI and luxR of four described category (separated by comma)*.

† *Strains sequenced in this study*.

# *Topology variation and conservation of phyH in convergent double luxR, luxI gene neighborhoods (See **Figure 5B** for detailed topology variation)*.

### LuxR phylogeny reveals diverse origin of sphingomonad LuxR and supports the monophyletic clustering of LuxR solos from plant associated bacteria

**Figure 3:**
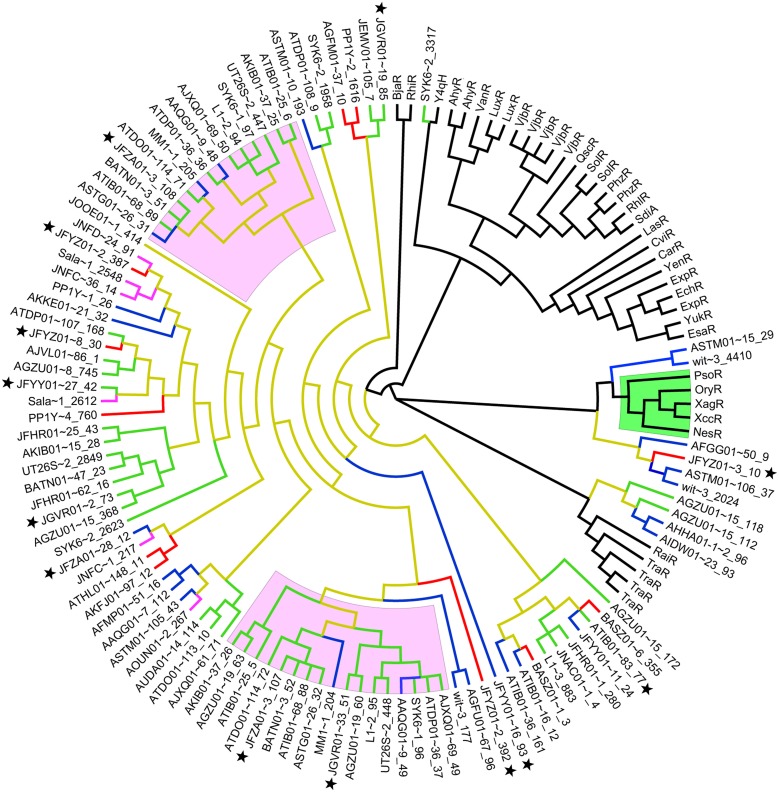
**Unrooted phylogenetic tree of functionally validated LuxR homologs, Plant associated bacteria (PAB) LuxR Solos (Gonzalez and Venturi, 2013) and Identified sphingomonad LuxR Homologs**. Clades highlighted in green and pink represent PAB LuxR solos and sphingomonad double LuxR, LuxI, respectively. Branches colored in brown, blue, red, green, and purple represent the *Sphingomonadaceae, Sphingomonas, Novosphingobium, Sphingobium*, and *Sphingopyxis* lineages, respectively. Black star next to taxa name indicates LuxR homologs from strains sequenced in this study. Accession numbers and aligned sequences are available in Supplemental Data 1.

**Figure 4:**
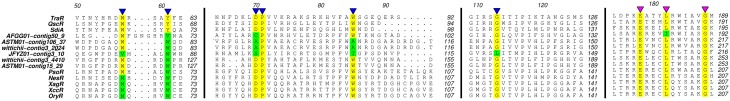
**Alignment of plant associated bacteria solos**. LuxR solos and selected sphingomonad LuxR homologs. Number above the alignment corresponds to the residue number of the TraR protein. Regions highlighted in yellow indicate the invariant sites of canonical LuxR homologs (Fuqua and Greenberg, 2002) while variation from the conserved site was highlighted in green. The conserved sites corresponding to autoinducer binding and DNA binding were indicated by blue and purple triangles, respectively.

A majority of the sphingomonad LuxR homologs form a big clade that is a sister group to the clade containing the functionally validated BjaR and RhlR (Figure 3) (Cubo et al., 1992; Lindemann et al., 2011). Consistent with previous reports, the PAB LuxR solos e.g., NesR, XagR, XccR, OryR, and PsoR (Ferluga et al., 2007; Zhang et al., 2007; Ferluga and Venturi, 2009; Chatnaparat et al., 2012; Gonzalez et al., 2013) formed a robust and well-defined monophyletic group. Based on phylogenetic clustering, six sphingomonad LuxR homologs may share a common (but distant) ancestry with the PAB LuxR solos clade. Alignment of these six putative LuxR homologs shows substitution in the highly conserved amino acid in the regulatory domain e.g., Y61W that is similarly reported in PAB LuxR solos. With the exception of a LuxR homolog from *Sphingobium herbicidovorans* NRBC 16415 (JFYZ01~contig3_10) that has a W57V substitution, the W57 residue was conserved in the remaining five sphingomonad LuxR homologs. Furthermore, other substitutions were observed in the conserved D70 and W85 residues for four out of the six sphingomonad LuxR homologs (Figure 4). In general, the three conserved residues in the DNA-binding domain (E178, L182, and G188) are conserved across the LuxR homologs alignment with the exception of L182I substitution in a *Sphingomonas* sp. S17 LuxR homolog (AFGG01~contig50_9).

### The gene neighborhood of sphingomonad *LuxR* solo and *LuxR* double is not conserved

**Figure 5:**
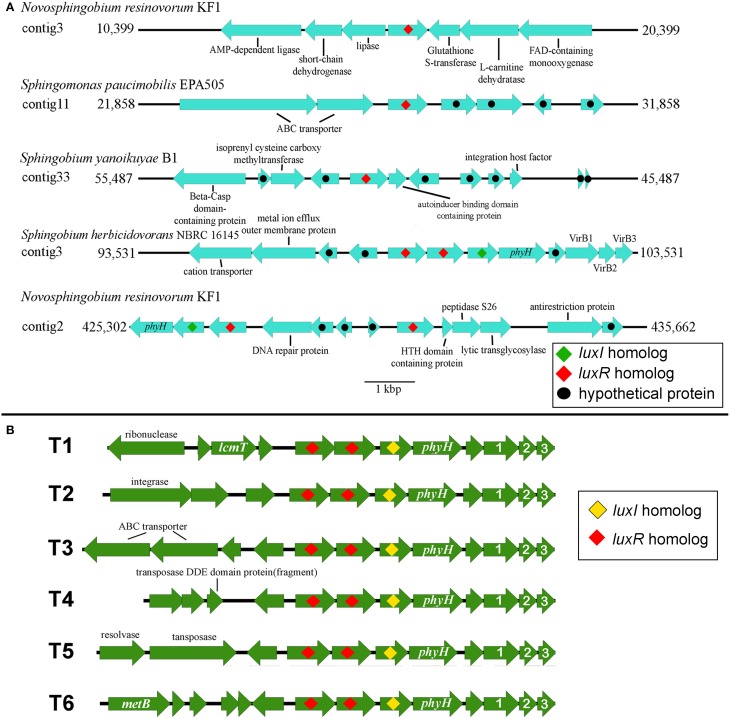
**Gene organization of *luxR* Solos and *luxR-luxR-luxI* in the four whole genome sequenced sphingomonads**. **(A)** EasyFig generated linear comparison of *luxR* solos in the genomic region of selected sphingomonads. Approximately 5000 bp of genomic region flanking the *luxR* solos is shown. **(B)** Gene orientation of the identified convergent double *luxR, luxI* group and its gene neighborhood variation. T1 to T6 denotes different gene neighborhoods identified in the convergent double *luxR, luxI*. Arrows without label represent gene coding for hypothetical protein. Please see Table 2 for topology variation present in sphingomonad genomes. The numbers “1,” “2,” and “3” represent *virB1, virB2*, and *virB3* genes respectively. Additional abbreviations include: *lcmT*, Isoprenylcysteine carboxyl methyltransferase; *metB*, Cystathionine gamma-synthase; *phyH*, phytanoly dioxygenase.

Investigation of the genes flanking the putative *luxR* solos in our sequenced genomes reveals some intriguing findings (Figure 5A). In *Sphingobium herbicidovorans* NBRC 16415, its putative *luxR* solo is convergently oriented with respect to a *luxI/R* pair and while in *N. resinovorum* [contig 2], it is located four genes downstream of a *luxI/R* pair. Furthermore, the gene coding for a possibly truncated LuxR-like protein is located immediately downstream of the *luxR* solos in *S. yanoikuyae* and *N. resinovorum* (contig2) (Figure 5A), suggesting the occurrence of *luxR* gene duplication and/or recombination in that region. In addition to the tandem *luxR* duplication (*luxR* double) in strain NBRC16415, further analysis of the sphingomonad genomes led to the identification of additional tandem *luxR* duplication (Table 3 and Figure 3) with variable gene neighborhood at the 5′ end (Figure 5B).

### Pairwise comparison between members of the same convergent double *LuxR* group shows considerable sequence divergence

**Figure 6:**
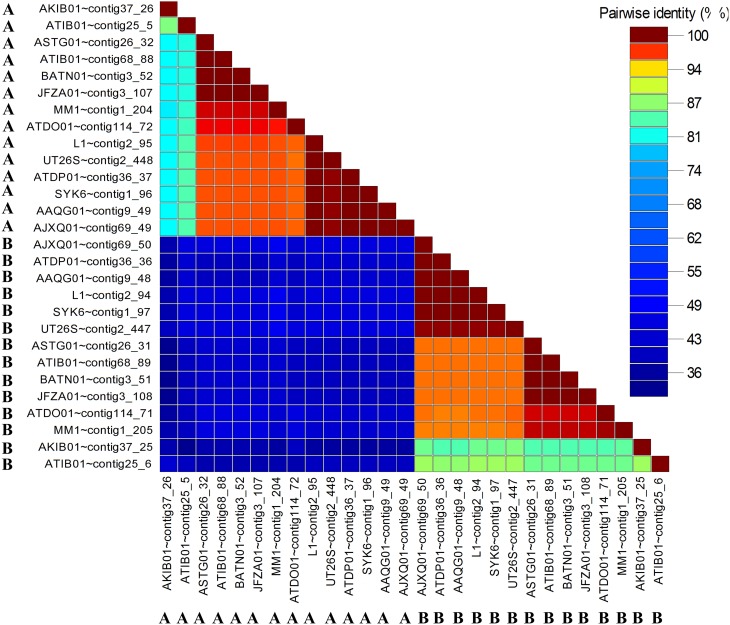
**Pairwise identity matrix of identified convergent luxR-luxR-luxI (LuxR-A and LuxR-B) in sphingomonads**. The letters A and B correspond to different partner *luxRs* in the *luxR-luxR-luxI*. The genes coding for LuxR homologs with the same symbol and color were convergently oriented with respect to each other.

The amino acid pairwise identity between members of the same LuxR double group is in the range of 50%. On the contrary, up to 94% pairwise identity could be obtained for members from different LuxR double group (Figure 6). This is consistent with the LuxR phylogenetic tree with whereby LuxR double members from the same group do not form a tight cluster with one another (Figure 3). Given that *luxR* double is almost exclusively observed in the genus *Sphingobium, luxR* double may originate from an ancient tandem gene duplication in the common ancestor of the genus *Sphingobium* followed by a neofunctionalization-oriented functional divergence of the *luxR* duplicate that was subsequently retained in several strains of the genus *Sphingobium*. The presence of a *luxR* double in a non-*Sphingobium* strain e.g., *Sphingomonas sp*. MM1 may then be attributed to horizontal gene transfer.

### Identification of LuxI solos

**Figure 7:**
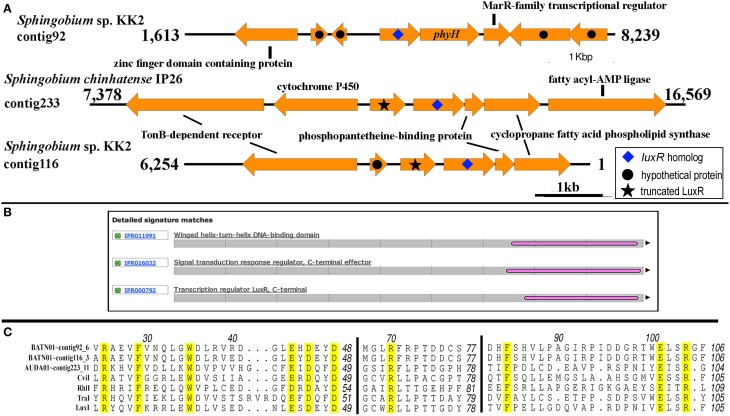
**Genomic and genetic evidence for the presence of *luxI* solos in sphingomonads**. **(A)** Gene neighborhood showing non-*LuxR* genes located in the vicinity of the putative LuxI genes (arrow with blue diamond). Analysis of the translated protein sequence for the gene upstream and convergently oriented to the putative luxI gene in *Sphingobium chinhatense* IP26 and *Sphingobium* sp. KK2 indicates that it may be an N-terminal truncated LuxR protein (arrow with black star). **(B)** A representative Interproscan analysis domain analysis of the N-terminal truncated LuxR protein noted in Figure 7A. **(C)** Protein alignment of the putative LuxI solos. Number above the alignment corresponds to the amino acid residue of TraI. Amino acid residues are conserved in all LuxI-type proteins (Fuqua and Greenberg, 2002) are highlighted in yellow.

Two putative *luxI* solos were identified in *Sphingobium* sp. KK2 strain that reside on different contigs and one in *Sphingobium chinhatense* IP26 strains (Figure 7A). A gene coding for N-terminal truncated/mutated LuxR-like protein is located immediately upstream and convergently oriented to the putative *luxI* solo in *Sphingobium chinhatense* IP26 and *Sphingobium* sp. KK2. Multiple sequence alignment of the three putative LuxI solos in sphingomonads with LuxI-type family proteins showed all 10 amino acid residues required for AHL synthase activity are conserved and supports that these three *luxI* solos encode enzymes involved in AHL synthesis (Figure 7B). Additionally, *phyH* gene coding for phytanoyl dioxygenase is located immediately downstream of and convergently oriented with respect to one of the *luxI* solos in strain KK2 which is frequently observed in several well-described *luxI/R* pairs (Gan et al., 2013).

## Discussion

The biochemical and genetic characterization of *Novosphingobium* sp. Rr 2-17 isolated from grapevine tumor provided the first glimpse of QS ability in the genus *Novosphingobium* (Gan et al., 2009). The genome sequencing of strain Rr 2-17 and subsequent comparative genomic analysis with five additional members from the genus *Novosphingobium* validates the presence of *luxI/R* homolog(s) (Gan et al., 2012) and even more intriguingly, a *luxR* solo in this genus (Gan et al., 2013). Expanding from our previous study, we present four new whole genome sequences of AHL QS signal producing strains in the sphingomonad group and to our knowledge presents the most comprehensive genomic surveillance of sphingomonads for the distribution of *luxI/R* homologs to date. In addition, the work presents the most updated and accurate genome-based taxonomy validation of the currently sequenced sphingomonads. Although previous works provided convincing biochemical test results to support the reclassification of *Sphingomonas*, the constructed phylogeny based on the 16S rRNA gene failed to provide satisfactory bootstrap support particularly in the splits that separated the major genus in *Sphingomonadaceae* (Takeuchi et al., 2001). Our phylogenomic approach dramatically improves the bootstrap support at these major splits that highlights the presence of strong phylogenetic signal afforded by the utilization of nearly 400 universal proteins. Further, the paraphyletic clustering of the genus *Sphingomonas* underscores the overlooked diversity of *Sphingomonas* that may benefit from further sub-classification in addition to its current classification into three well-known genera e.g., *Novosphingobium, Sphingopyxis*, and *Sphingobium* and the recently proposed genus, *Sphingosinicella* (Takeuchi et al., 2001).

The phylogenetic clustering of sphingomonad LuxR homologs shows no evidence of phylogeny congruence i.e., inconsistent clustering of LuxR homologs from members of the same genus. Given that a majority of the sphingomonad LuxR homologs form a large clade among themselves, the incongruence with the newly constructed species phylogeny (Figure 2) can be explained by a combination of horizontal gene transfer and gene duplication within the *Sphingomonadaceae* family followed by speciation as proposed previously (Lerat and Moran, 2004). Interestingly, four sphingomonad LuxR homologs formed a monophyletic clade that is sister group to TraR and RaiR. The distant relationship between the this sphingomonad LuxR clade and the major sphingomonad LuxR homologs clade coupled with the localization of both *traR* and *raiR* genes on the plasmid e.g., Ti plasmid and non-symbiotic plasmid respectively (Piper et al., 1993; Gray et al., 1996; Oger and Farrand, 2002) suggest the acquisition of these four *luxR* homologs via plasmid-mediated horizontal gene transfer. This warrants future work focusing on the identification of plasmid-coded *luxR* homolog through plasmid isolation and sequencing to confirm the origin of the distant sphingomonad *luxR* homologs.

Five out of six of the sphingomonad LuxR homologs that are more closely related to PAB LuxR solos than the rest of the LuxR homologs (Figure 3) appear to share one of the two major signature e.g., Y61W in PAB solos (Figure 4). Recent cartography analysis of the ligand-binding sites of the LuxR homologs has demonstrated that Y61 residue is directly involved in ligand binding (in addition to W57, D70, and W85) (Covaceuszach et al., 2013). Therefore, substitution at Y61 in these specific sphingomonad LuxR homologs is a strong indicator of their inability to bind to AHL. Three dimensional structure modeling of these proteins followed by comparison of binding/active sites regarding substrate preference(s) will shed lights into the protein characteristic of these atypical sphingomonad LuxR homologs. Recently, a LuxR-homolog from *Photorhabdus* that has some substitutions in the conserved 9 aa residues in LuxR homologs was shown to bind to a bacterial-produced pyrone instead of AHLs or plant exudates (Brachmann et al., 2013; Brameyer et al., 2014). It should be noted that the structural-activity relationship(s) of LuxR solos is beyond the scope of this study.

The occurrence of two *luxR* homologs in tandem is not novel in the realm of alpha-bacteria and has been previously reported in the genus *Roseobacter*, noted as topology N (Cude and Buchan, 2013). However, the gene neighborhood of the double *luxR* in various sphingomonads is significantly different from topology N to justify the proposal of a new topology that we will coin as topology T. Topology T represents the convergently oriented *luxR-luxR-luxI*-*phyH*-X-*virB1*-*virB2*-*virB3* topology whereby X denotes gene coding for hypothetical protein. It is also worth noting that one or more mobile elements are present upstream of the double *luxR* in three out of the six topology variants, indicating past transposition event(s) and/or transposition potential of the gene cluster.

The low pairwise identity between members of the same LuxR double group (Figure 6) support the distantly shared ancestry as observed in the LuxR phylogenetic tree (Figure 3, shaded in pink). Furthermore, the low pairwise identity between members of the same LuxR double group and retention of convergent double *luxR* in the genomes of several *Sphingobium* strains suggests that the sphingomonad LuxR duplicate has undergone sufficient functional divergence which may correlate to the evolution of the organism to be more competitive regarding niche adaptation. The presence of the complete LuxR signature domains in both members of the same convergent double LuxR group suggests the retention of the core LuxR function with perhaps dissimilar substrate range and/or DNA-binding region that warrants future protein characterization and transcription study.

In addition to harboring the newly described QS gene circuit arrangement, some members of the currently sequenced genus *Sphingobium* exhibit another interesting feature of QS signaling, e.g., presence of *luxI* solos. The assignment of LuxI solos based on the presence of signature amino acid residues in canonical LuxI homologs and the absence of unassociated *luxR* in the vicinity of its protein coding gene (Figure 7 and Table 3) provide strong evidence that the LuxI solos identified in both *Sphingobium* sp. KK2 and *Sphingobium chinhatense* IP26 are authentic. The presence of a gene coding for a putative N-terminal truncated LuxR-like protein immediately upstream of the *luxI* solo gene in strain IP26 is suggestive of the *luxI* solo previously being part of a functional *luxI/R* pair instead of having been acquired independently. However, this may not be the case for another *luxI* solo located in contig92 of *Sphingobium* sp. KK2 with more than 800 bp of an upstream non-protein coding region. Recently a detailed study of LuxR-LuxI type QS network in *Ruegeria* sp. KLH11 (Zan et al., 2012) confirmed the presence of a functional LuxI solo, SscI and demonstrated that SscI and a paired-LuxI homolog, SsbI, produced the same AHLs e.g., 3-OH-C14:1-HSL and 3-OH-C14-HSL that indirectly affect QS-dependent gene regulation by another LuxI/R pair homologs, SsaI/R. Given the presence of one or more *luxI/R* pairs in the strain KK2 and IP26 genomes, it is tempting to speculate that a similar level of QS network complexity may operate in both *Sphingobium* strains.

### Conflict of interest statement

The authors declare that the research was conducted in the absence of any commercial or financial relationships that could be construed as a potential conflict of interest.
