# Supplementary material for: Whole genome sequencing and analysis reveal insights into the genetic structure, diversity and evolutionary relatedness of luxI and luxR homologs in bacteria belonging to the Sphingomonadaceae family
Source: Front Cell Infect Microbiol. 2015 Jan 8;4:188. doi: 10.3389/fcimb.2014.00188 (PMC4288048; doi:10.3389/fcimb.2014.00188)
Supplement: Supplementary file 1 [file Table1.PDF]

Supplemental Table 1.

N-acyl-homoserine lactone bacterial biosensor strains used in this work.

**Biosensor strain**	**Receptor**	**Cognate AHL***
A. tumefaciens A136 (pCF218, pMV26)	TraR	3-oxo-C8-HSL
C. violaceum CV026	CviR	C6-HSL
E. coli JM109 (pSB401)	LuxR	3-oxo-C6-HSL
E. coli JM109 (pSB536	AhyR	C4-HSL
E. coli JM109 (pSB1075)	LasR	3-oxo-C12-HSL

* C4-HSL, *N*-butanoyl-homoserine lactone; C6-HSL, *N*-hexanoyl-homoserine lactone; 3-oxo-C6-HSL, *N*-3-oxo-hexanoyl-homoserine lactone; 3-oxo-C8-HSL, *N*-3-oxo-octanoyl-homoserine lactone; 3-oxo-C12-HSL, *N*-oxo-dodecanoyl-homoserine lactone.
