# Supplementary material for: Whole genome sequencing and analysis reveal insights into the genetic structure, diversity and evolutionary relatedness of luxI and luxR homologs in bacteria belonging to the Sphingomonadaceae family
Source: Front Cell Infect Microbiol. 2015 Jan 8;4:188. doi: 10.3389/fcimb.2014.00188 (PMC4288048; doi:10.3389/fcimb.2014.00188)
Supplement: Supplementary file 5 [file DataSheet1.DOCX]

>Novo_AGFM01~contig37_10 # 13065 # 13832 # -1 # ID=37_10;partial=00;start_type=ATG;rbs_motif=GGAG/GAGG;rbs_spacer=5-10bp;gc_cont=0.585

----------------------------------------------MVI-----------

-----------------------------------GPELVEFML---------SLGQVRT

DADFYDLLLETTRRLGFKQFAFVSHVD------LLAAADEAVAISNYPDGWV---ERILA

ERYYLDDPVHAASIGRSTPYAWHSIQRRVR-------LSKRQLSIMQEGASFGL----QD

GVTVPV-HSPGEYRGT-CSFATSERVSM-----TPRIRGATHIVAGFGFEAARKLVRIRL

----GAEKSTPSLPRLSPREVDCVGLVATGMGDTQIAHALGLSEATVHQHVTGAMRKCGV

FKRTALVFRALFDGHICFHSLRRTSSK----------------

>Novo_AKFJ01~contig97_12 # 12938 # 13690 # 1 # ID=97_12;partial=00;start_type=ATG;rbs_motif=GGA/GAG/AGG;rbs_spacer=11-12bp;gc_cont=0.600

----------------------------------------------MGI-----------

-----------------------------------TGAYQEFRD---------LVFEAKN

LTALGHALSRIAQALECDYYAVGHHVD------WGRRTPNAFRLQNYPARWV---EFYDR

SGFGGRDPVHRASARTQSGFLWREAGHLID-------FTASDARLMALAMRHGI----AD

GFTVPA-NVPGEFQGS-CTFATAPDNPL-----TEERVMLARLIAPHIFEAGRRLVGNG-

-----QLIRNARTARLTHRQRTCLLWIAAGKSDWETGQILGISEGTVRQHISDSCKRLGI

QKRTLLLFLAFRNGAISFPEVPFH-------------------

>Novo_AKKE01~contig21_32 # 37680 # 38330 # 1 # ID=21_32;partial=00;start_type=ATG;rbs_motif=GGA/GAG/AGG;rbs_spacer=11-12bp;gc_cont=0.618

------------------------------------------------------------

------------------------------------------------------------

--------------MGYDHFALTYDDR------PGHADRRSLLIHDYPDEWA---EVYTG

FSLSRADPIRRGAECSFTGFAWQRIGDIIP-------LTASDWRMLAVGRDNGI----DD

GYTVPR-HLPGEASGS-CSFVVRPGRRL-----PHHMLGATELVGALALTTARRIAGI--

-------RPPSDRPVLTDRQCECVLWSARGKTAEEIAGILGIKPDTVVQHLKAARERYDV

HSRSSLILCVLFDGLISFGDIFRWWR-----------------

>Novo_ATHL01~contig148_11 # 11723 # 12496 # -1 # ID=148_11;partial=00;start_type=ATG;rbs_motif=GGA/GAG/AGG;rbs_spacer=11-12bp;gc_cont=0.605

----------------------------------------------MGV-----------

-----------------------------------NTAYRKFKE---------IVKATQD

LESLELVLDKITQAVGCDYYAIGHHID------WGSGKPQAFRLLNYPEAWI---EFYDR

NGFFGKDFVHRASQRTQNPFLWREAAEFVE-------YSTNDERFLDLARQYGV----DD

GFTVPS-NVPGEFHGS-CTFATVPGRPL-----DEDGLVLARLIAPEVFAAARRLVGLA-

-----EVMECINSPLLTERQRECLIWMMAGKTDLETGIILGISHQTVRRHLNEAGRRLGA

LNRPLLAFLACRSGVISYPEVPCGAYTRNRA------------

>Novo_BASZ01~contig1_3 # 5030 # 5758 # -1 # ID=1_3;partial=00;start_type=ATG;rbs_motif=AGGAG;rbs_spacer=5-10bp;gc_cont=0.582

----------------------------------------------MYV-----------

-----------------------------------GPHLRKALS---------RLNRASS

IEALRDGLADAARQMGFPFVALVQHGG------LPRLVERSMVVTNYPAEFV---QSYIE

NHYFIIDPVYEVSYQLDRPFGWDEITNYVE-------LADHQHALFEEAQGYGI----IH

GITVPL-HIPSETYAS-CTFSRPTPIAI-----TPSLMTTLQIVAGFAFKTGLYLHHA--

-------MQGRNVPRLTRREAECTALIALGKTDWEIGEILGIAQTSVRYFISRAKQRYGV

FRRSELVARAIVDAQIPMN------------------------

>Novo_BASZ01~contig6_355 # 359850 # 360641 # -1 # ID=6_355;partial=00;start_type=ATG;rbs_motif=AGGAG;rbs_spacer=5-10bp;gc_cont=0.604

----------------------------------------------MYV-----------

-----------------------------------NARMRETLS---------RFNAASS

MEALLAALADAATKMGFPYVAMIQHGG------LPRLVERALVITNYPAEFV---RFYTE

SHAYVIDPVYEVSQLLDRPFSWDEIPGYVD-------LTGTQSALFEEARLHGL----VH

GVTVPL-HIPSESHAS-CTFARAEPIMA-----SPSLLTTLHIVAAFGFKAGLRLHHA--

-------SRGHDVPRLTRREAQCTALVAVGKSDWEISQILGLSETSVRYFVSHAKQRYGV

YKRSELVARALIDAQILRNDQGIIEAGPRRPRHRAKRRS---P

>Novo_JFYZ01.1~contig2_387 # 426890 # 427642 # -1 # ID=2_387;partial=00;start_type=GTG;rbs_motif=4Base/6BMM;rbs_spacer=13-15bp;gc_cont=0.649

----------------------------------------------MKQ-----------

-----------------------------------YYLTEELAF---------EITNALT

QDELFTALSAAAERLGFDHFALAYDRR-------GGSTPASLLVHDYPAAWA---QVYVD

FDLGGADPVRRAGERSMTGFEWRSLPDLIP-------LTKGDRQMLEVGRENGI----AD

GFTVPR-HLPGEASGA-CSFVIGPNSRM-----QEEVLHVAEIVGAVALSAARRLVGV--

-------APPKGRPTLSERQRECVLWTARGKTASEVGAILGISEETVIQHLKTARDRYDV

HCRQMLILCALFDGLIGFADIYDWWHDH---------------

>Novo_JFYZ01.1~contig2_392 # 430662 # 431405 # 1 # ID=2_392;partial=00;start_type=GTG;rbs_motif=GGA/GAG/AGG;rbs_spacer=5-10bp;gc_cont=0.649

----------------------------------------------MPL-----------

-----------------------------------ERLIEQHRK---------YVRAARR

DRDILSLTVDAVGELGFNRVALVQMVW------FLRQERQYFCLDNY-GEWH---DIFIA

RQYYRRDPVHLASLRTNRCFAWSEVGAILG-------ASRVHLPILQEAARHGL----NR

GLTVPI-GVPGEPPGS-GSLGTDAETLP-----PPDRCRAAAWIVDEAFAEVRRVYGLP-

------AKAEDHAPPLSPRRLECLRLVALGLTDAEVAERMGIAISTVHTHMEYLRRTYGV

RSRTQLGRLAQRLGLLGTEDIIP--------------------

>Novo_JFYZ01.1~contig3_10 # 15399 # 16196 # 1 # ID=3_10;partial=00;start_type=ATG;rbs_motif=None;rbs_spacer=None;gc_cont=0.607

----------------------------------------------MLSTP---------

----------------------------RNPQGAVADIATDALHHGLA-----ELDAASD

FAAAQVGLKAVAVAIGMPLLAWAPDVSRPE---FDEHMDAFLRQEGWPDEVM---ALWWN

RNAMLKSPLYIRCRTSGMPFVTGPSDNVPP-------RTAELRQIVSAINAMGV----RS

LITMPI-HLPRGRVAM-VTWGGSATKAL-ARSVLAQTRTTLIAAAHLFMSSYLHENAGF-

------RSSEEELARLTPREWQCLRLTAQGFREEQVATTIGLGSTTVRFHLDNVVQKLGA

ANRTHAVALAAQLGLLGS--------------------I---G

>Novo_JFYZ01.1~contig8_30 # 67808 # 68572 # 1 # ID=8_30;partial=00;start_type=ATG;rbs_motif=GGA/GAG/AGG;rbs_spacer=5-10bp;gc_cont=0.609

----------------------------------------------MS------------

-----------------------------------FEWIESLGE---------LIIKAGS

ADELREIMAKATHDLGFDRFALSLEIG------CGSEFGTSVLIHDYPASWA---DLYIG

FNLAETDPIRRAAERSLLGFKWKRVRHLIP-------VTDFERRTLEAGRKHGI----VD

GYTVPR-HIPGEITGS-CSFVTGPGRTL-----PEALLPGAELLGAIALASARNVSGW--

-------QERSEAPKITDRQRDCVLWAARGKTDWEISRILGISRATVVQHLREARDRYDA

DKRASLILCALFDGLISFADIFRWRERRQGRR-----------

>Novo_Novosphingobium_PP1Y~contig1_26 # 21431 # 22204 # 1 # ID=1_26;partial=00;start_type=ATG;rbs_motif=GGA/GAG/AGG;rbs_spacer=5-10bp;gc_cont=0.592

----------------------------------------------MR------------

-----------------------------------INLAEKLMS---------EVSGASS

RSALFTALENATRHMSFDHFALVYDDC------RASNENDAFLVHDYPPEWA---RVYQS

LGLATQDPVRRTCERSFTSFPWGVSREFTG-------AVRGDHKILGLSREYGI----GD

GFTVPR-HLPGGVTGS-CTFVVRPDAAL-----PVEMLQVADLLGGVALASARKIAWL--

-------HMSASSPGLTDRQRECLLWWGRGKTAAEIAIIMGLSVETVHQHLKLARERYGV

DASQSVLACAIAEGLIGPGDIWRWFRTRPGIAVTG--------

>Novo_Novosphingobium_PP1Y~contig2_1616 # 1737074 # 1737841 # -1 # ID=2_1616;partial=00;start_type=ATG;rbs_motif=GGA/GAG/AGG;rbs_spacer=5-10bp;gc_cont=0.600

----------------------------------------------MFI-----------

-----------------------------------GSELIEFLL---------CLGQVRS

DADFFDLMLETTRRLGFEQFAFVSHVD------LVAASEDAVAISNYPDGWV---ERILT

ERYYLDDPVHAASIGRNTPYAWHAIGTEVI-------QSKRQRTILKEGASFGL----QD

GITVPV-HSPGEYRGT-CSFATSQPVSM-----TPQIRGATHIVAGFGFETARKLVRLRL

----GLEQTVPTLPRFSPREVDCVGLVASGMGDTQIAHALGLSEATVHQHITAAMRKCGV

FKRAALVFRSLFDGHICFHSLRRTSSK----------------

>Novo_Novosphingobium_PP1Y~contig4_760 # 875522 # 876235 # -1 # ID=4_760;partial=00;start_type=ATG;rbs_motif=None;rbs_spacer=None;gc_cont=0.630

----------------------------------------------M-------------

-------------------------------------LAAELAD---------TFTHSRS

GDELHEALARSAHEMGFDHFALALEVG------GCAGNGISVLIHDYPASWA---DAYVD

FNLAASDPVRRAAERSVLGFKSCGIGDLIP-------VTEAEQSMFNIGRRHGV----AD

GYTVPQ-QVPDDLTGS-CSFVTAPSRNL-----PEAMLAVAELVGAVAIASGRRVAGW--

-------EDPVERPRLTDHQRDYVLWAARGKTDWETSKILGISHETVIQHLKEARERSDT

TKRASLIIFAT-------------------------PRRGEWI

>Pyxis_AOUN01~contig2_267 # 294663 # 295433 # -1 # ID=2_267;partial=00;start_type=ATG;rbs_motif=GGA/GAG/AGG;rbs_spacer=5-10bp;gc_cont=0.628

----------------------------------------------MPRSTMASE-----

-----------------------------------SQFAEDFLA---------AVRATAS

ESELCALMVAATRELGFRHYALIHHSA------PSDDSFGRVDMKDYPQAVE---KRLFQ

EGHFRRDPILRACLFADSAFLWSDLDRYID-------LDRRDKASLAFGLALGL----NQ

GITVPC-VLLGDCIGS-CTFAGTRHPDL-----AQRRLGVVQVAGIFAFQAARRLSGAA-

-------HRRFPPPQLHPRPRDCVVLAGRGLSNKEIARALALSPRTVDGYLTEAREMFGA

HDRTELVVSAVLAGEVGLHELRR-QPE----------------

>Pyxis_JNFC~contig1_217 # 252663 # 253406 # 1 # ID=1_217;partial=00;start_type=ATG;rbs_motif=None;rbs_spacer=None;gc_cont=0.648

----------------------------------------------MPT-----------

-----------------------------------MDAAHAFAL---------DVTRVKD

AAGLADLLAEACARMGCSWFALSHHVD------FLAAPDRGVRVHNYPEDWA---RWFDE

RGLGLTDPVHRASHRSLEGFFWRNMKPLSG-------ERPEDELVLSEAQRHGI----GD

GLTIPA-HIPGEAHGS-VSFAWTPGIAA-----NDMALLFARMIGGPAFEAARLLANP--

-------ELAQVGPRLTDRQRECLILSAKGNSAPKVGRILDLSPDTVREHLRNARQRYDA

NGGITLTVRALYAGDLSYEDIAKR-------------------

>Pyxis_JNFC~contig36_14 # 11344 # 12099 # 1 # ID=36_14;partial=00;start_type=GTG;rbs_motif=3Base/5BMM;rbs_spacer=13-15bp;gc_cont=0.642

----------------------------------------------MKH-----------

-----------------------------------FNLTEELAH---------DISGADK

EERLFAALSNAADRMGFDLFALAFDRR------GGGGEGASMLVHNYPDAWA---NVYVG

FDLSGTDPIRRAGEKSMTGFQWRNVDHYIP-------LSRGDRQLLKVARESGI----GD

GFTVPR-HLPGEATGS-CSFAVAPNARI-----PLEMLHAAEIVGAVALAAARQLIGT--

-------GSYTPRAALTERQRECVLWSARGKTAGETASILGISEETVVRHLKIARERYSV

PCRQMLILCALFDGVIGFSDVYDWWRPL---------------

>Pyxis_JNFD~contig24_91 # 88672 # 89424 # -1 # ID=24_91;partial=00;start_type=GTG;rbs_motif=GGA/GAG/AGG;rbs_spacer=5-10bp;gc_cont=0.628

----------------------------------------------MKY-----------

-----------------------------------FDLMEELAL---------EISTATN

EDGLSTALAQASLRMGFDHFALAYDRR-------GRAEPASLLVHNYPDAWA---KVYVG

FDLGGADPVRRAGERSMTGFRWGELESYIP-------LTRGDRQMLHVGREYGL----AD

GYTIPR-HLPGEASGA-CSFVLRPRSEF-----RDDMLRIAEIIGAIAIVTARQLVGA--

-------VPPKGRATLSERQRECVLWTARGKTAAEVALILGIGEETVVQHLKTARDRYDV

HCGQMLTLCALFDGLIGFADVYDWWHLP---------------

>Pyxis_Sphingopyxis_alaskensis~contig1_2548 # 2661727 # 2662488 # 1 # ID=1_2548;partial=00;start_type=ATG;rbs_motif=None;rbs_spacer=None;gc_cont=0.626

MND-------------------------------------------VKH-----------

-----------------------------------YYLTEELAQ---------EFTGADR

EEQLFAALTKAAGRMGFDHFALAFDRR-------GTGEPASILVHNYPDAWA---KVYVG

FDLSGTDPIRRASERSLTGFEWRHVDRYIP-------LSRGDRQLLSVARDSGV----GD

GFTVPR-HLPGEASGT-CSFAVRPHADI-----PADMLHAAEILGAIAIASARELIGS--

-------SPLRPRPILTERQRECVLWSARGKTAGEIADILGISEETVVRHLKMARERYSV

HCRSMLILCALFDGLIGFSDIYDWWRPN---------------

>Pyxis_Sphingopyxis_alaskensis~contig1_2612 # 2731109 # 2731861 # 1 # ID=1_2612;partial=00;start_type=ATG;rbs_motif=AGxAGG/AGGxGG;rbs_spacer=5-10bp;gc_cont=0.588

----------------------------------------------MR------------

-----------------------------------LGLVELLGD---------SILTASS

IDHLHEALTRATRDMGFDRFALSLEIG------CGADSSTSLLIHDYPASWA---DVYIG

FNLAASDPVRRAAERSVLGFGWRNILDLVP-------MTEIEKTTFETGRRHGL----AD

GFTVPR-HLPGDVTGS-CSFVTGLGKSI-----PHAMLIVAEMLGAMAIASARQLSGW--

-------TSRSVQPRLTDRQRDCVLWAARGKTDWEISRILDISHETVIQHLKDARERYET

HKRASLILYALYDGLISFADIFRWRVRS---------------

>Sphingobium_AGZU01~contig8_745 # 807127 # 807879 # 1 # ID=8_745;partial=00;start_type=ATG;rbs_motif=None;rbs_spacer=None;gc_cont=0.612

----------------------------------------------MN------------

-----------------------------------QEQIESLSK---------TILRALS

ADALHEAMDRIAIALGFDRFALSVDVG------LGGHSGTSMLVHSYPASWA---DICIG

FNLAQTDPVRRAGESSLSGFRWREIEHLIP-------ITPIERVTFETGRKHGM----VD

GFTVPR-HLPGTVTGS-CTFVTATERPL-----PERMLIIADILGAIAIAQASRLSGW--

-------RRPIKKPRLTDRQRDCVLWAARGKTNWEIGRILGISKETVIQHLKEARDRYDT

SNRASLILYALFDGLISFSDIFRWRERV---------------

>Sphingobium_AGZU01~contig15_112 # 130624 # 131433 # -1 # ID=15_112;partial=00;start_type=ATG;rbs_motif=GGxGG;rbs_spacer=5-10bp;gc_cont=0.590

----------------------------------------------MFTNKLASNRGHDA

-----------------KTVVEASTLFLRPG----NANHPDILA---------TLKASRS

LSRLRDWLLDYARLLGFYGARYIHVGNFWTNEPDSSPHHPLRFLTTSPR------DADDA

DDWLVRDPCAAQVRTAIAPFAYSTRTKAG--------LDPIQRIWLENERARGV----SA

GIIIPV-QDSIQGPAY-ISLFGNDETGS--RDMAERCGPDLAFAAAHFHAKAKQFVPLA-

----------DWVPRLSAREQQVLRLASMGYTYAQSGEALGLSEKAIDYHLRNASDKLGA

QSKLRAVVLAFAHGLATV-------------------------

>Sphingobium_AGZU01~contig15_118 # 136737 # 137537 # 1 # ID=15_118;partial=00;start_type=ATG;rbs_motif=AGxAGG/AGGxGG;rbs_spacer=5-10bp;gc_cont=0.578

----------------------------------------------MLAGGALQRQ----

--------------------------FDHACVALDVSWVRHFVMEGGSVMAIGPIEDCAS

SVDLENWLEQLASEMGFDGARYHHVGHRPQG-GRTAKRPPLRFLST---------LENGQ

EPWRAGDPALSQIVQSFLPFVWSTKDDLS--------LPDLQRAWLSIERLRGV----EA

GVVIPV-QDYLSGPAY-ISLFSEKLSQA--ISAVEQRRHEMATLAIEFHLRAKQLIT---

-------IRTRSSVLLSDREFSCLRNAAAGVALAESAGSLGISVRTVETHLAKATLKLGG

TNRINAVAIAIGSGMIHV-------------------------

>Sphingobium_AGZU01~contig15_172 # 213319 # 214062 # 1 # ID=15_172;partial=00;start_type=GTG;rbs_motif=AGGAG;rbs_spacer=5-10bp;gc_cont=0.565

----------------------------------------------MFI-----------

-----------------------------------GPHLRDILS---------QILKATS

FDSLCESLARGAKQMGFDHVALVQHGN------LPRLAERALVVTNYPAEFI---NSYIE

NHHYVFDPVYEVSELLDRPFSWDEIPAIVS-------LREPQLALFGAARVHGL----SH

GVTVPL-HVPGEPRAS-CTFAASRVVEA-----TPDLLAALHVIASFGFNMALRLYQP--

-------ARRNAGPKLTRREAECTTLVALGKTDWEIGKILGLGGATVKYFISVAMQRYGV

YKRSALVARALMDGQILREVNDSE-------------------

>Sphingobium_AGZU01~contig15_368 # 425228 # 426004 # -1 # ID=15_368;partial=00;start_type=ATG;rbs_motif=None;rbs_spacer=None;gc_cont=0.616

----------------------------------------------MEPD----------

-----------------------------------YFLLQQLTR---------QVQDAAT

LEDYREAMAALTQGLNFDYFALTHHVD------PRSAGEEAVHLHNYPAQWA---DYYAR

HALGICDPIHRASHVAPGGFPWARIGDYID-------LTARDRHMLRLGASQGI----GD

GFTIPI-RIEGEMPGS-CTFAMRSGRPL-----DSAWLALAEIAGRFAFDGARRLIGRQ-

-----ALRLPMLGRILTRRQRDCLLWAFRGKSDWEISQILKVTEGTVKRHILNACARYRV

NKRIMLLAPTLLDGTFSITEIYGYRDTSFGT------------

>Sphingobium_AGZU01~contig19_60 # 55449 # 56210 # 1 # ID=19_60;partial=00;start_type=ATG;rbs_motif=GGA/GAG/AGG;rbs_spacer=11-12bp;gc_cont=0.639

M---------------------------------------------ANRIKDCGR-----

-----------------------------------LDDLTRFAA---------DCQRAET

LGDLHGTIDAAVRDLGFRWFTLLHNID------LRRGGEQSLFLTTYPSAWL---EEVLE

ERHYLEDPIHAACARTPSGLAWDRIGDVLE-------LTPRQRSILDRARDHDL----AS

GYSLPI-RTPGEPEAI-FTAARSRDEPL-----SAEEVLTARLLGSVAYDRARELLGER-

-------AGPLACVPLSPRQVECIALVAQGKSDWEIGQILGLSRDTVHEYVESARRRYGV

RRRTQLVLRAVRDGHLNMEALL---------------------

>Sphingobium_AGZU01~contig19_63 # 58250 # 59008 # 1 # ID=19_63;partial=00;start_type=ATG;rbs_motif=GGA/GAG/AGG;rbs_spacer=5-10bp;gc_cont=0.598

----------------------------------------------MQGTRDIGR-----

-----------------------------------LATIGAFEQ---------SSKLATT

LDQLSNLVDAIVRDFGFRWFALLHNVD------LVQRSKNALMLTTYPVKWL---DEVIE

TRLYLDDPVHAAVGKTPSGLNWNQIGEFID-------PSTRQIATLAKGRDHGL----RQ

GFTMPI-RMADEPDAL-FSVARANDDEI-----SGEDALAARLIGTIAFDRGRAILGEE-

-------RLSYEPVPLSPRQIDCIALVGQGKSDWEAAQILGLSRDTVHEYVEGARRRYGV

RRRTQLVLRAVRDGHLNIDAML---------------------

>Sphingobium_AJVL01~contig86_1 # 1264 # 2016 # 1 # ID=86_1;partial=00;start_type=ATG;rbs_motif=3Base/5BMM;rbs_spacer=13-15bp;gc_cont=0.643

----------------------------------------------MY------------

-----------------------------------HEQIETLSQ---------TIIRALS

ADALHEAMDRIALALGFDRFALSVEVG------LGGPSGTSMLLHSYPPSWA---DVYIG

FNLAHTDPVRRAGESSLSGFRWRDIEALIP-------MTPIERVTFETGRRHGM----VD

GFTVPR-HLPGTVTGS-CTFVTGADRPL-----PERMLAIADILGAIAIAQASRLSGW--

-------CRTAKKPRLTDRQRDCVLWAARGKTNWEIARILGISKETVIQHLKEARDRYDT

SNRASLILYALFDGLISFSDVFRWRERL---------------

>Sphingobium_AJXQ01~contig61_71 # 75746 # 76426 # -1 # ID=61_71;partial=00;start_type=ATG;rbs_motif=None;rbs_spacer=None;gc_cont=0.636

------------------------------------------------------------

------------------------------------------------------------

------MMEVIAREMGFRHFALAHHDN------RVESEAGRVRLMDYPAAVT---DRLVE

QGLFRRDPVVRACLHAVTAFLWSDLPDMLT-------LDRGDRESLAFGAREGL----NE

GITVPF-VHLGDAIGS-CTFAGTNRPDR-----SGIYLGAAQMIGVFAFQAARRLVGAA-

-------TPIPRSPRLHPRPRDCVVLAGRGLSNKEIARALALTPRTVDGYLTAARRLFGV

HDRTELVITAVLAGEVGLDELRRRQPE----------------

>Sphingobium_AJXQ01~contig69_49 # 44842 # 45603 # 1 # ID=69_49;partial=00;start_type=ATG;rbs_motif=GGA/GAG/AGG;rbs_spacer=11-12bp;gc_cont=0.636

M---------------------------------------------ANRIKDCGR-----

-----------------------------------LDDLTRFAT---------DCQRAET

LGDLHGTIDAAVRDLGFRWFTLLHNID------LRRGDEQSLFLTTYPSAWL---EEVLE

ERHYLEDPIHAACARTPSGLAWDRVGDVLE-------LTPRQRGILDRARDHDL----AS

GYSLPI-RTPGEPEAI-FTAARSRDEPL-----SAEEVLTARLLGSVAYDRARELLGER-

-------AGPLACVPLSPRQVECIALVAQGKSDWEIGQILGLSRDTVHEYVESARRRYGV

RRRTQLVLRAVRDGHLNMEALL---------------------

>Sphingobium_AJXQ01~contig69_50 # 45686 # 46363 # 1 # ID=69_50;partial=00;start_type=TTG;rbs_motif=AGGA;rbs_spacer=5-10bp;gc_cont=0.631

------------------------------------------------------------

------------------------------------------------------------

---MSDLMAEITKAMGFRHYALVHHVD-------LKPAVRSVHIVDYPQDWV---ERFQA

RRLYASDPIHRASHRTNVGFAWSAVQSIIS-------LTAADRSILAEAYEAGL----GD

GFTVPA-HIPGELNGS-CSFAMAAGEML-----DHRQLPFVQLVGSFAFEAARKISRIH-

-------APQSECPSLTERQAECVALVARGKTDWEISQILGIGQETVIQHVKDARDRYGV

TKRTLLAIRALFEGQISFADVFGR-------------------

>Sphingobium_AKIB01~contig15_28 # 36014 # 36775 # -1 # ID=15_28;partial=00;start_type=ATG;rbs_motif=GGA/GAG/AGG;rbs_spacer=5-10bp;gc_cont=0.631

------------------------------------------------------------

-------------------------------------MATQFRR---------VVDAVAN

GDDFAAAMKGLTDQLGFQYFALTHHVD------LAKASGGAIHLHNYPTQWA---DYYHR

HALGVTDPVHRASQVARVGFPWSHMAEMIP-------LSGRDRRILALGRAQGI----GD

GFTVPAVHVPGEARGS-CSFANEAGRQL-----PHDMLPLAEVAGLFAFEGARRLWSIG-

-----SLRQAPPHPMLTDRQRDCVLWVARGKSDWEISLILKVGEETVARHIKQACERYGV

NKRTYLVILTLFDGTLTFSDIFRRRYYPFPE------------

>Sphingobium_AKIB01~contig37_25 # 21667 # 22419 # -1 # ID=37_25;partial=00;start_type=TTG;rbs_motif=GGA/GAG/AGG;rbs_spacer=5-10bp;gc_cont=0.600

----------------------------------------------MAIR----------

-----------------------------------LAEVRSFCD---------SVSAAKS

PDALYDFMQDITARMGFAHFALVHHVD------TAVPATGTIRLVDYPQSWM---DIFEE

RRLYAADPIHRASQQTSVGFAWNNVEALIA-------LSPRDRAVLTAARDVGI----GD

GFTIPA-HIPGEINGS-CSFATASGVGL-----DEEQLPLVQLVGNFAFEAARRVNRSV-

------GSHPRELPRLTDRQVECVALVARGKTDWEIARILGVGSETVSQHLKDARDRYGV

TKRTMLAMRGLFDGSISFADVFGR-------------------

>Sphingobium_AKIB01~contig37_26 # 22431 # 23189 # -1 # ID=37_26;partial=00;start_type=ATG;rbs_motif=GGAG/GAGG;rbs_spacer=5-10bp;gc_cont=0.580

----------------------------------------------MASKCDKGR-----

-----------------------------------LADISVFEH---------QAKSTTT

FDQLSSIVDSFVRQVGFRWFALLHNVD------LVQRSKKAIMLTTYPVRWL---DEIIE

TRLYLDDPIHAACAKTSSGLTWDRIDEFIV-------PSPHQVSILKKGSAHGL----RT

GFTMPI-RMQDEPEAV-FTIARSTDAVA-----TTEDVLTARLVGTVAFDQARAILGDE-

-------GIVYQPVSLSPRQIDCIALVAQGKSDWEISQILSLGRDTVHEYVEGARRRYGV

RRRTQLVLRAVRDGHLNINALL---------------------

>Sphingobium_ASTG01~contig26_31 # 24954 # 25697 # -1 # ID=26_31;partial=00;start_type=ATG;rbs_motif=None;rbs_spacer=None;gc_cont=0.605

----------------------------------------------MIG-----------

-----------------------------------LGQVQDFCA---------VAAIIKD

SQALAGLMAEITRAMGFRYVSLVHHID-------LKPAVSSVHIVDYPPEWV---ERFQA

RRLYASDPIHRASHRTNIGFAWSAVQSLIT-------LSAADRSILAEAHDAGL----GD

GFTVPA-HIPGEVNGS-CSFAMGSGDVL-----DQRQLPLVQLIGSFAFEAARRLSRQT-

-------PAPCEGPCLTERQAECVALVARGKTDWEISQILGIGQETVIQHVKDARDRYGV

TKRTLLAIRALFDGQISFADVFGR-------------------

>Sphingobium_ASTG01~contig26_32 # 25714 # 26475 # -1 # ID=26_32;partial=00;start_type=ATG;rbs_motif=GGA/GAG/AGG;rbs_spacer=11-12bp;gc_cont=0.640

M---------------------------------------------ANRIRDCGR-----

-----------------------------------LDDLTRFAQ---------ECARAGN

LGDLQGTIDAAVRELGFRWFTLLHNVD------LRHGGGQRLFLTTYPPAWL---EEVLE

ERHYIEDPIHAACARTPSGLAWDRVGDVLE-------LSSRQRSILKRARDHDL----AA

GYSLPI-RTPGEPEAI-FTVARPRDEPL-----DAAEILTARLLGSVAYDRARELLGEE-

-------IRTLAFVSLSPRQVECIAFVAQGKSDWEIAQILGLSRDTVHEYVESARRRYGV

RRRTQLVLRAVRDGHLNMDALL---------------------

>Sphingobium_ATDO01~contig113_10 # 10697 # 11377 # -1 # ID=113_10;partial=00;start_type=ATG;rbs_motif=None;rbs_spacer=None;gc_cont=0.636

------------------------------------------------------------

------------------------------------------------------------

------MMEVIAREMGFRHFALAHHDN------RVESEAGRVRLMDYPAAVT---DRLVE

QGLFRRDPVVRACLHAVTAFLWSDLPDMLT-------LDRGDRESLAFGAREGL----NE

GITVPF-VHLGDAIGS-CTFAGTNRPDR-----SGIYLGAAQMIGVFAFQAARRLVGAA-

-------TPIPRSPRLHPRPRDCVVLAGRGLSNKEIARALALTPRTVDGYLTAARRLFGL

HDRTELVITAVLAGEVGLDELRRRQPE----------------

>Sphingobium_ATDO01~contig114_71 # 71333 # 72076 # -1 # ID=114_71;partial=00;start_type=ATG;rbs_motif=None;rbs_spacer=None;gc_cont=0.614

----------------------------------------------MIW-----------

-----------------------------------LGQVQEFCA---------IAATIKD

SRALAGLMAEITREMGFRYFALVHHID-------LKPAVSSVHIVDYPPEWV---ERFQA

RRLYASDPIHRASHRTNIGFAWSAVQSLIT-------LSAADRSILAEAHDAGL----GD

GFTVPA-HIPGEVNGS-CSFAMGAGDVL-----DQRQLPLVQLIGSFAFEAARRLSRQT-

-------QAPCEGPSLTERQAECVALVARGKTDWEISPILGIGQETVIQHVKDARDRYGV

TKRTLLAIRALFDGQISFADVFGR-------------------

>Sphingobium_ATDO01~contig114_72 # 72093 # 72854 # -1 # ID=114_72;partial=00;start_type=ATG;rbs_motif=GGA/GAG/AGG;rbs_spacer=11-12bp;gc_cont=0.634

M---------------------------------------------TNWITDCGR-----

-----------------------------------LADLTRFAE---------ECERAES

LGDLRGTIDATVRELGFRWFTLLHNVD------LRRGGGQRLFLTTYPPAWL---EEVLE

ERHYVEDPIHAACARTPSGLAWDRVGDVLE-------LSSRQRSILQRARDHDL----AA

GYSLPI-RMLGEPEAI-FTVARPRDEPL-----DAAEILTARLLGSVAYDRARQLLGEE-

-------MRTLAFVSLSPRQVECIALVAQGKSDWEIAQILGLSRDTVHEYVESARRRYGV

RRRTQLVLRAVRDGHLNMDALL---------------------

>Sphingobium_ATDP01~contig36_36 # 42571 # 43317 # -1 # ID=36_36;partial=00;start_type=ATG;rbs_motif=None;rbs_spacer=None;gc_cont=0.627

---------------------------------------------MMIG-----------

-----------------------------------LGQVQDFCA---------VAGAIKD

ATALSDLMAEITKAMGFRHYALVHHVD-------LKPAVRSVHIVDYPQDWV---ERFQA

RRLYASDPIHRASHRTNVGFAWSAVQSIIS-------LTAADRSILAEAYEAGL----GD

GFTVPA-HIPGELNGS-CSFAMAAGEML-----DHRQLPFVQLVGSFAFEAARKISRIH-

-------APQSECPSLTERQAECVALVARGKTDWEISQILGIGQETVIQHVKDARDRYGV

TKRTLLAIRALFEGQISFADVFGR-------------------

>Sphingobium_ATDP01~contig36_37 # 43331 # 44092 # -1 # ID=36_37;partial=00;start_type=ATG;rbs_motif=GGA/GAG/AGG;rbs_spacer=11-12bp;gc_cont=0.636

M---------------------------------------------ANRIKDCGR-----

-----------------------------------LDDLTRFAT---------DCQRAET

LGDLHGTIDAAVRDLGFRWFTLLHNID------LRRGDEQSLFLTTYPSAWL---EEVLE

ERHYLEDPIHAACARTPSGLAWDRVGDVLE-------LTPRQRGILDRARDHDL----AS

GYSLPI-RTPGEPEAI-FTAARSRDEPL-----SAEEVLTARLLGSVAYDRARELLGER-

-------AGPLACVPLSPRQVECIALVAQGKSDWEIGQILGLSRDTVHEYVESARRRYGV

RRRTQLVLRAVRDGHLNMEALL---------------------

>Sphingobium_ATDP01~contig107_168 # 157400 # 158164 # -1 # ID=107_168;partial=00;start_type=ATG;rbs_motif=AGxAG;rbs_spacer=5-10bp;gc_cont=0.587

----------------------------------------------MY------------

-----------------------------------FELIESLGE---------LILAANS

VDELHELLAKTTHDLGFDRFALSLEIG------CGSAFGTSVLIHDYPASWA---DMYIG

FNLAETDPIRRAAERSLLGFKWQRIRHLIP-------VTDFERRTFEAGHKYGI----VD

GYTVPR-HMPGEITGS-CSFVIGTDRNL-----PEAMLPGAELLGAIALASARNVSGY--

-------EERSEPPKLTDRQRDCVLWAARGKTDWEISRILGISRDTVVQHLREARDRYDA

DKRASLILCALFDGLISFADIFRWRERRKRRR-----------

>Sphingobium_ATDP01~contig108_9 # 7242 # 7997 # -1 # ID=108_9;partial=00;start_type=ATG;rbs_motif=AGGAG;rbs_spacer=5-10bp;gc_cont=0.554

----------------------------------------------MLH-----------

-----------------------------------SQEYQSIIE---------AFYEAKD

NQEIQGVLEHLTYFLGFRHFAIGHHVD------LLSPPSTSFGISNYTPGWL---SEVFH

EGYYMDDPIHFLCNGRNTGFIWPDPHLLNR-------LNERHRHILERGALRNF----RA

GYTIPV-HLPGEYSGS-CTFATPLSGDI-----SREVLPAAFYAASHAFEAMRRQARMA-

-----AGLSVACPPEITPRQREIVLLLGQGKSYAEMGDILGISRNTAHQHCKTVFRSYGN

IQRCNLIARVLFDGLASFPEMLRKH------------------

>Sphingobium_ATIB01~contig16_12 # 21923 # 22651 # -1 # ID=16_12;partial=00;start_type=ATG;rbs_motif=AGGAG;rbs_spacer=5-10bp;gc_cont=0.582

----------------------------------------------MYV-----------

-----------------------------------GPHLRKALS---------RLNRASS

IEALRDGLADAARQMGFPFVALVQHGG------LPRLVERSMVVTNYPAEFV---QSYIE

NHYFIIDPVYEVSYQLDRPFGWDEITNYVE-------LADHQHALFEEAQGYGI----IH

GITVPL-HIPSETYAS-CTFSRPTPIAI-----TPSLMTTLQIVAGFAFKTGLYLHHA--

-------MQGRNVPRLTRREAECTALIALGKTDWEIGEILGIAQTSVRYFISRAKQRYGV

FRRSELVARAIVDAQIPMN------------------------

>Sphingobium_ATIB01~contig25_5 # 6707 # 7531 # 1 # ID=25_5;partial=00;start_type=TTG;rbs_motif=GGAG/GAGG;rbs_spacer=5-10bp;gc_cont=0.595

M-----------------------FFNGFAEWWLDDAVIEMRTVLIMGSLKDAGR-----

-----------------------------------LGDITAFEA---------ECRQATT

LEQLKSKLDAIVRQFGFRWFALVHNVD------LKRTTRKALLITTYPVRWI---EEVMD

ARLYMEDPVHAACAKTVSGLTWDQIGDFIA-------PNARQLSILERGRAHGL----AA

GFTMPI-RMRDEPDAI-FTVARQGDEMI-----SSPDLLSARLVGTVAFDRARTLLGPE-

--------LANVPIALSPRQIDCLDLVAQGKSDWEIGQILGLSRDTVHEYVEGARRRYGV

RRRTQLVLRAVRDGHLNIEALV---------------------

>Sphingobium_ATIB01~contig25_6 # 7552 # 8301 # 1 # ID=25_6;partial=00;start_type=ATG;rbs_motif=AGxAG;rbs_spacer=11-12bp;gc_cont=0.613

----------------------------------------------MIT-----------

-----------------------------------LAEVQAFCT---------VARAATT

PHALMQAVEEITAAMGFRYFALVHHVD------LLTPGSTIVRLVSYPRDWV---DAFEE

GRLYAADPIHRASHTTTVGFAWSKVATLIT-------LNPRDHAVLAAARTAGL----GD

GFTIPA-HIPGEANGS-CSFAMRTGEEL-----EDAQLPLVQLVGSFAFEAARKIARSG-

------IAPIPPAPQLTDRQTECVALVARGKTDWEIARILGVGVETVTQHLKDARDRYGV

TKRTMLAIRALFDGAISFTDILRR-------------------

>Sphingobium_ATIB01~contig36_161 # 132187 # 132963 # 1 # ID=36_161;partial=00;start_type=ATG;rbs_motif=AGGAG;rbs_spacer=5-10bp;gc_cont=0.625

----------------------------------------------MYI-----------

-----------------------------------GPRLREALS---------RFNTASS

TEALRDGLAGAAEQMGFPYVALVQHGG------LPRLVERSMVVTNYPAEFV---QSYIE

NHYFIIDPVYDVSQQLDRPFGWEEITSFVE-------LADHQHALFGEARRYGI----VH

GITVPL-HIPSESYAS-CSFARPEPIEI-----TPSLMATLQIVAGFAFKAGLYLHHA--

-------MHGRNVPRLTRREAECTALIALGKTDWEIGQILGIAQTTVRYFISRAKQRYGV

FRRSELVARAIVDAQVIRDGQDKVRESAGDKPERE--------

>Sphingobium_ATIB01~contig68_88 # 80949 # 81710 # 1 # ID=68_88;partial=00;start_type=ATG;rbs_motif=GGA/GAG/AGG;rbs_spacer=11-12bp;gc_cont=0.640

M---------------------------------------------ANRIRDCGR-----

-----------------------------------LDDLTRFAQ---------ECARAGN

LGDLQGTIDAAVRELGFRWFTLLHNVD------LRHGGGQRLFLTTYPPAWL---EEVLE

ERHYIEDPIHAACARTPSGLAWDRVGDVLE-------LSSRQRSILKRARDHDL----AA

GYSLPI-RTPGEPEAI-FTVARPRDEPL-----DAAEILTARLLGSVAYDRARELLGEE-

-------IRTLAFVSLSPRQVECIAFVAQGKSDWEIAQILGLSRDTVHEYVESARRRYGV

RRRTQLVLRAVRDGHLNMDALL---------------------

>Sphingobium_ATIB01~contig68_89 # 81727 # 82470 # 1 # ID=68_89;partial=00;start_type=ATG;rbs_motif=None;rbs_spacer=None;gc_cont=0.605

----------------------------------------------MIG-----------

-----------------------------------LGQVQDFCA---------VAAIIKD

SQALAGLMAEITRAMGFRYVSLVHHID-------LKPAVSSVHIVDYPPEWV---ERFQA

RRLYASDPIHRASHRTNIGFAWSAVQSLIT-------LSAADRSILAEAHDAGL----GD

GFTVPA-HIPGEVNGS-CSFAMGSGDVL-----DQRQLPLVQLIGSFAFEAARRLSRQT-

-------PAPCEGPCLTERQAECVALVARGKTDWEISQILGIGQETVIQHVKDARDRYGV

TKRTLLAIRALFDGQISFADVFGR-------------------

>Sphingobium_ATIB01~contig83_77 # 78212 # 79003 # 1 # ID=83_77;partial=00;start_type=ATG;rbs_motif=AGGAG;rbs_spacer=5-10bp;gc_cont=0.602

----------------------------------------------MYV-----------

-----------------------------------NARMRETLS---------RFNAASS

MEALLAALADAATKMGFPYVAMIQHGG------LPRLVERALVITNYPAEFV---RFYTE

SHAYVIDPVYEVSQLLDRPFSWDEIPGYVD-------LTGTQSALFEEARLHGL----VH

GVTVPL-HIPSESHAS-CTFARAEPIMA-----SPSLLTTLHIVAAFGFKAGLRLHHA--

-------SRGHDVPRLTRREAQCTALVAVGKSDWEISQILGLSETSVRYFVSHAKQRYGV

YKRSELVARALIDAQILRNDQGIIEAGPRRPRHRAKRRS---P

>Sphingobium_AUDA01~contig14_114 # 117786 # 118466 # 1 # ID=14_114;partial=00;start_type=ATG;rbs_motif=None;rbs_spacer=None;gc_cont=0.634

------------------------------------------------------------

------------------------------------------------------------

------MMEVIAREMGFRHFALAHHDN------RVESEAGRVRLMDYPAAVT---DRLVE

QGLFRRDPVVRACLHAVTAFLWSDLPDMLT-------LDRGDRESLAFGAREGL----NE

GITVPF-VHLGDAIGS-CTFAGTNRPDR-----SGIYLGAAQMIGVFAFQAARRLVGAA-

-------TPIPRSPRLHPRPRDCVVLAGRGLSNKEIARALALTPRTVDGYLTAARRLFGL

HDRTELVITAVLAGEVGLDELRRRQPE----------------

>Sphingobium_BATN01~contig3_51 # 50002 # 50745 # -1 # ID=3_51;partial=00;start_type=ATG;rbs_motif=None;rbs_spacer=None;gc_cont=0.603

----------------------------------------------MIG-----------

-----------------------------------LGQVQDFCA---------VAAIIKD

SQALAGLMAEITRAMGFRYVSLVHHID-------LKPAVSSVHIVDYPPEWV---ERFQA

RRLYASDPIHRASHRTNIGFAWSAVQSLIT-------LSAADRSILAEAHDAGL----GD

GFTVPA-HIPGEVNGS-CSFAMGSGDVL-----DQRQLPLVQLIGSFAFEAARRLSRQT-

-------PAPCEGPCLTERQAECVALVARGKTDWEISQILGIGQETVIQHVKDARDRYGV

TKRTLLAIRALFDGQISFADVFGR-------------------

>Sphingobium_BATN01~contig3_52 # 50762 # 51523 # -1 # ID=3_52;partial=00;start_type=ATG;rbs_motif=GGA/GAG/AGG;rbs_spacer=11-12bp;gc_cont=0.640

M---------------------------------------------ANRIRDCGR-----

-----------------------------------LDDLTRFAQ---------ECARAGN

LGDLQGTIDAAVRELGFRWFTLLHNVD------LRHGGGQRLFLTTYPPAWL---EEVLE

ERHYIEDPIHAACARTPSGLAWDRVGDVLE-------LSSRQRSILKRARDHDL----AA

GYSLPI-RTPGEPEAI-FTVARPRDEPL-----DAAEILTARLLGSVAYDRARELLGEE-

-------IRTLAFVSLSPRQVECIAFVAQGKSDWEIAQILGLSRDTVHEYVESARRRYGV

RRRTQLVLRAVRDGHLNMDALL---------------------

>Sphingobium_BATN01~contig47_23 # 26970 # 27770 # 1 # ID=47_23;partial=00;start_type=TTG;rbs_motif=AGGA/GGAG/GAGG;rbs_spacer=11-12bp;gc_cont=0.624

M----------------------------------RCRLRPGRV-RMSM-----------

-----------------------------------HQEAMHFLQ---------CINQVRT

RDDMGSVMRVIAEQLGFQYFALTQHVD------VVAAGSGVIHIHNYPDRWA---DFYAA

NALGITDPVHRACHMTSWGFRWTQMPALIP-------LTSGDHRHLARGRQAGI----GD

GFTIPS-NIGGQPPGS-CTFANAGDRPL-----REDRLLLAQLLGNYAFDAARRLWSMP-

-------RQAMTAPLITDRQRDCVQWAARGKSDWETSQILDISRETVTSHIKEACARYQV

NKRILLVGRTLGDGTLTISDIGGWSHPHFWE------------

>Sphingobium_JEMV01~contig105_7 # 5017 # 5778 # 1 # ID=105_7;partial=00;start_type=ATG;rbs_motif=AGGAG;rbs_spacer=5-10bp;gc_cont=0.577

----------------------------------------------MYM-----------

-----------------------------------SPRLATFVD---------DCREVTD

EFALKALLGEATPSLGFEQFALVDHVD------LIGPPHDTMVITSYPEGWI---ERSLL

RNYYVDDPVHAASTKTVTAFLWSVIPAMIR-------MTKRQRHILEEAKPFGL----RE

GLTIPV-QAAGEYRGS-CSFAGRETVRP-----TADLLGAAQVIGMFAFEAARRLQRARW

----APGTKTPDIPALSQRELDCIALVGCGKGNAEIGGILNISPNTVRQYIEDAMRKYDV

YKRTELVVRALFDGQICYRALNIRR------------------

>Sphingobium_JFHR01~contig1_280 # 289933 # 290724 # 1 # ID=1_280;partial=00;start_type=ATG;rbs_motif=AGGAG;rbs_spacer=5-10bp;gc_cont=0.602

----------------------------------------------MYV-----------

-----------------------------------NARLRETLS---------RFNVASS

MEALLAALADAATKMGFPYVAMIQHGG------LPRLVERALVITNYPAEFV---RFYTE

SHAYVIDPVYEVSQLLDRPFSWDEIPGYVD-------LTGTQSALFEEARLHGL----VH

GVTVPL-HIPSESHAS-CTFARAEPIMA-----SPSLLTTLHIVAAFGFKAGLRLHHA--

-------SRGHDVPRLTRREAQCTALVAVGKSDWEISQILGLSETSVRYFVSHAKQRYGV

YKRSELVARALIDAQILRNDQGIIEAGPRRPRHRAKRRS---P

>Sphingobium_JFHR01~contig25_43 # 45342 # 46115 # 1 # ID=25_43;partial=00;start_type=ATG;rbs_motif=None;rbs_spacer=None;gc_cont=0.661

----------------------------------------------MGM-----------

-----------------------------------HNMATQFMR---------VVEAVAN

ADDLAAAMTAVTEQLGFQYFALTHHVD------IIAASGSAIRLHNYPARWA---DYYDR

NALGVSDPVHRASHMTSVGFPWSRMSDLIP-------MTREDHRVLAMGRDQGI----GD

GFTVPA-HVPGEARGS-CSFANEAGRPM-----PNEMLPLAQLAGMFAFEGARRLWAMR-

-----GGLQMPPRPVLTDRQRDCVLWVARGKSDWEISLILGVGEETVARHIKQACERYGV

NKRTYLVILTLFDGTLTFSDIFRRRYYPFPE------------

>Sphingobium_JFHR01~contig62_16 # 14805 # 15575 # -1 # ID=62_16;partial=00;start_type=ATG;rbs_motif=None;rbs_spacer=None;gc_cont=0.597

----------------------------------------------MSV-----------

-----------------------------------QYNVQEFLN---------DIERVQN

PDDMAMVLSDIAGTMGFQYFAITQHVD------VLAANGMAIHIHNYPAHWA---DFYAA

NALGLSDPVHRACHMTDWGFRWTSISSLIP-------LTRNDKAHLDCGRREGI----GD

GFTVPS-NVGGYPPGS-CSFANADGVPI-----PDDKLWLAQLVGSCAFHVARRLWLGA-

------VRRGTRNAALTDRQRECVQWMALGKNDDEAGEILGIRKGTVTKHIKDSCSRYEV

NKRVMLIGLALADGTLTISDIGGWRHSHLWE------------

>Sphingobium_JFZA01~contig3_107 # 98531 # 99292 # 1 # ID=3_107;partial=00;start_type=ATG;rbs_motif=GGA/GAG/AGG;rbs_spacer=11-12bp;gc_cont=0.640

M---------------------------------------------ANRIRDCGR-----

-----------------------------------LDDLTRFAQ---------ECARAGN

LGDLQGTIDAAVRELGFRWFTLLHNVD------LRHGGGQRLFLTTYPPAWL---EEVLE

ERHYIEDPIHAACARTPSGLAWDRVGDVLE-------LSSRQRSILKRARDHDL----AA

GYSLPI-RTPGEPEAI-FTVARPRDEPL-----DAAEILTARLLGSVAYDRARELLGEE-

-------IRTLAFVSLSPRQVECIAFVAQGKSDWEIAQILGLSRDTVHEYVESARRRYGV

RRRTQLVLRAVRDGHLNMDALL---------------------

>Sphingobium_JFZA01~contig3_108 # 99309 # 100052 # 1 # ID=3_108;partial=00;start_type=ATG;rbs_motif=None;rbs_spacer=None;gc_cont=0.603

----------------------------------------------MIG-----------

-----------------------------------LGQVQDFCA---------VAAIIKD

SQALAGLMAEITRAMGFRYVSLVHHID-------LKPAVSSVHIVDYPPEWV---ERFQA

RRLYASDPIHRASHRTNIGFAWSAVQSLIT-------LSAADRSILAEAHDAGL----GD

GFTVPA-HIPGEVNGS-CSFAMGSGDVL-----DQRQLPLVQLIGSFAFEAARRLSRQT-

-------PAPCEGPCLTERQAECVALVARGKTDWEISQILGIGQETVIQHVKDARDRYGV

TKRTLLAIRALFDGQISFADVFGR-------------------

>Sphingobium_JFZA01~contig28_12 # 13316 # 14110 # -1 # ID=28_12;partial=00;start_type=ATG;rbs_motif=GGA/GAG/AGG;rbs_spacer=5-10bp;gc_cont=0.626

MPGKQ----------------------------VRECCRRCHGS-FMPT-----------

-----------------------------------LDAALRLAA---------ESLKVAN

EDALIDILDEACSRLGCSWFALSHHVD------FLAAPEKGLRVHNYPEDWA---RWFDE

QRLGVRDPVHRASARRAAGFLWHEMHHFSR-------PRPGDGAILKRARRHGI----HD

GLTVPT-HIPGDAHGS-VSFAWQKGHYA-----TPEALMFAQVIGAFVFEAARQLSGL--

-------VPANDQPRLTDRQIECLLWAARGKSDSVIAQIMDLQTDTVSEHLRNARAKYSA

RSRVSLAIRALFDGTICFSDVAGG-------------------

>Sphingobium_JGVR01~contig2_73 # 81909 # 82685 # -1 # ID=2_73;partial=00;start_type=ATG;rbs_motif=None;rbs_spacer=None;gc_cont=0.616

----------------------------------------------MEPD----------

-----------------------------------YYLLQQLTR---------QVQDAAT

LEDYREAMAALAQGLNFDYFALTHHVD------PRSAGEQTVHLHNYPAQWA---DYYAR

HALGICDPIHRASHVAPGGFPWARIGDYID-------LTARDRHMLRLGASQGI----GD

GFTIPI-RIEGEMPGS-CTFAMRSGRPL-----DSAWLAFAEIAGRFAFDGARRLMGRQ-

-----ALRLPMLGRILTRRQRDCLLWAFRGKSDWEISQILKVTEGTVKRHILNACARYRV

NKRIMLLAPTLLDGTFSITEIYGYRDTSFGT------------

>Sphingobium_JGVR01~contig19_85 # 76854 # 77612 # 1 # ID=19_85;partial=00;start_type=ATG;rbs_motif=AGGAG;rbs_spacer=5-10bp;gc_cont=0.594

----------------------------------------------MYI-----------

-----------------------------------SPRLGKFVN---------SCGEVQD

EFALKELLAEVTPSLGFDQFALVSHVD------LVGPPRDAMVITSYHEGWI---ERSLL

RNYYADDPVHAASTKTVTAFLWSVIPAMIR-------MTKRQQQILEEAKPFGL----RE

GLTIPV-QAPGEYRGT-CSFGGKNSVTL-----DADLRGAAQLVGMFAFEAARRLQRARW

----APGTQVPEIPTLSQRELDCIALVACGKGNSEIGGILKISPNTVRQYIDEAMRKYDV

YKRTELVVRALFDGQICYRALNID-------------------

>Sphingobium_JGVR01~contig33_51 # 50487 # 51248 # -1 # ID=33_51;partial=00;start_type=ATG;rbs_motif=GGA/GAG/AGG;rbs_spacer=11-12bp;gc_cont=0.639

M---------------------------------------------ANRIKDCGR-----

-----------------------------------LDDLTRFAA---------DCQRAET

LGDLHGTIDAAVRDLGFRWFTLLHNID------LRRGGEQSLFLTTYPSAWL---EEVLE

ERHYLEDPIHAACARTPSGLAWDRIGDVLE-------LTPRQRSILDRATDHDL----AS

GYSLPI-RTPGEPEAI-FTAARSRDEPL-----SAEEVLTARLLGSVAYDRARELLGER-

-------AGPLACVPLSPRQVECIALVAQGKSDWEIGQILGLSRDTVHEYVESARRRYGV

RRRTQLVLRAVRDGHLNMEALL---------------------

>Sphingobium_JNAC01~contig1_4 # 2701 # 3492 # -1 # ID=1_4;partial=00;start_type=ATG;rbs_motif=AGGAG;rbs_spacer=5-10bp;gc_cont=0.604

----------------------------------------------MYV-----------

-----------------------------------NARLRETLS---------RFNVASS

MEALLAALADAATKMGFPYVAMIQHGG------LPRLVERALVITNYPAEFV---RFYTE

SHAYVIDPVYEVSQLLDRPFSWDEIPGYVD-------LTGTQSALFEEARLHGL----VH

GVTVPL-HIPSESHAS-CTFARAEPIMA-----SPSLLTTLHIVAAFGFKAGLRLHHA--

-------SRGHDVPRLTRREAQCTALVAVGKSDWEISQILGLSETSVRYFVSHAKQRYGV

YKRSELVARALIDAQILRNDQGIIEAGPRRPRHRAKRRS---P

>Sphingobium_Sphingobium_L1~contig2_94 # 93843 # 94520 # -1 # ID=2_94;partial=00;start_type=TTG;rbs_motif=AGGA;rbs_spacer=5-10bp;gc_cont=0.633

------------------------------------------------------------

------------------------------------------------------------

---MSDLMAEITKAMGFRHYALVHHVD-------LKPAVRSVHIVDYPQDWV---ERFQA

RRLYASDPIHRASHRTNVGFAWSAVQSIIS-------LTAADRSILAEAYEAGL----GD

GFTVPA-HIPGELNGS-CSFAMAAGEML-----DHRQLPFVQLVGSFAFEAARKISRIH-

-------APQSECPSLTERQAECVALVARGKTDWEISQILGIGQETVIQHVKDARDRYGV

TKRTLLAIRALFEGQISFADVFGR-------------------

>Sphingobium_Sphingobium_L1~contig2_95 # 94603 # 95364 # -1 # ID=2_95;partial=00;start_type=ATG;rbs_motif=GGA/GAG/AGG;rbs_spacer=11-12bp;gc_cont=0.633

M---------------------------------------------ANRIKDCGR-----

-----------------------------------LDDLTRFAT---------DCQRAET

LGDLHGTIDAAVRDLGFRWFTLLHNID------LRRGDEQSLFLTTYPSAWL---EEVLE

ERHYIEDPIHAACARTPSGLAWDRVGDVLE-------LTPRQRGILDRARDHDL----AS

GYSLPI-RTPGEPEAI-FTAARSRDEPL-----SAEEVLTARLLGSVAYDRARELLGER-

-------AGPLACVPLSPRQVECIALVAQGKSDWEIGQILGLSRDTVHEYVESARRRYGV

RRRTQLVLRAVRDGHLNMEALL---------------------

>Sphingobium_Sphingobium_L1~contig3_883 # 969316 # 970107 # -1 # ID=3_883;partial=00;start_type=ATG;rbs_motif=AGGAG;rbs_spacer=5-10bp;gc_cont=0.604

----------------------------------------------MYV-----------

-----------------------------------NARLRETLS---------RFNVASS

MEALLAALADAATKMGFPYVAMIQHGG------LPRLVERALVITNYPAEFV---RFYTE

SHAYVIDPVYEVSQLLDRPFSWDEIPGYVD-------LTGTQSALFEEARLHGL----VH

GVTVPL-HIPSESHAS-CTFARAEPIMA-----SPSLLTTLHIVAAFGFKAGLRLHHA--

-------SRGHDVPRLTRREAQCTALVAVGKSDWEISQILGLSETSVRYFVSHAKQRYGV

YKRSELVARALIDAQILRNDQGIIEAGPRRPRHRAKRRS---P

>Sphingobium_Sphingobium_SYK6~contig1_96 # 92759 # 93520 # 1 # ID=1_96;partial=00;start_type=ATG;rbs_motif=GGA/GAG/AGG;rbs_spacer=11-12bp;gc_cont=0.636

M---------------------------------------------ANRIKDCGR-----

-----------------------------------LDDLTRFAT---------DCQRAET

LGDLHGTIDAAVRDLGFRWFTLLHNID------LRRGDEQSLFLTTYPSAWL---EEVLE

ERHYLEDPIHAACARTPSGLAWDRVGDVLE-------LTPRQRGILDRARDHDL----AS

GYSLPI-RTPGEPEAI-FTAARSRDEPL-----SAEEVLTARLLGSVAYDRARELLGER-

-------AGPLACVPLSPRQVECIALVAQGKSDWEIGQILGLSRDTVHEYVESARRRYGV

RRRTQLVLRAVRDGHLNMEALL---------------------

>Sphingobium_Sphingobium_SYK6~contig1_97 # 93603 # 94280 # 1 # ID=1_97;partial=00;start_type=TTG;rbs_motif=AGGA;rbs_spacer=5-10bp;gc_cont=0.631

------------------------------------------------------------

------------------------------------------------------------

---MSDLMAEITKAMGFRHYALVHHVD-------LKPAVRSVHIVDYPQDWV---ERFQA

RRLYASDPIHRASHRTNVGFAWSAVQSIIS-------LTAADRSILAEAYEAGL----GD

GFTVPA-HIPGELNGS-CSFAMAAGEML-----DHRQLPFVQLVGSFAFEAARKISRIH-

-------APQSECPSLTERQAECVALVARGKTDWEISQILGIGQETVIQHVKDARDRYGV

TKRTLLAIRALFEGQISFADVFGR-------------------

>Sphingobium_Sphingobium_SYK6~contig2_1958 # 2152248 # 2153003 # 1 # ID=2_1958;partial=00;start_type=ATG;rbs_motif=AGGAG;rbs_spacer=5-10bp;gc_cont=0.567

----------------------------------------------MLH-----------

-----------------------------------SQEYQSIIE---------AFYDARD

NQDIQGVLEHLTYFLGFRHFAIGHHVD------LLNPPSTSFGVSNYTPGWL---SEVFH

EGYYMDDPIHFLCNGRNTGFIWPDPHLLKR-------LNERHRHILERGALRNF----QA

GYTIPV-HLPGEYSGS-CTFATPLSGHI-----SREVLPAAFYAASHAFEAMRRQARMA-

-----AGLSVEPPPEITPRQREVVLLLGQGKSYAEMGDILGISGNTAHQHCKAVFRSYGN

IQRCNLIARVLFDGLASFPEMLRKH------------------

>Sphingobium_Sphingobium_SYK6~contig2_2623 # 2833586 # 2834332 # 1 # ID=2_2623;partial=00;start_type=ATG;rbs_motif=None;rbs_spacer=None;gc_cont=0.643

----------------------------------------------MLL-----------

-----------------------------------TRIAREFAD---------AVDEAQT

AEALATILHDVAREMGFHYFALTHHVD------VPLAPQPAIRLHNYPAAWV---DYFDA

HHLGLSDPVHRASQQTNVGFSWSRLAGMIE-------LTARDRDILERSQRAGI----GE

GFTVPA-HVPGESAGS-CSFAVAAGQPL-----REERLPLIQLVGAIAFEGARRVSALR-

-------MPAPARPRLTDRQRDCVYWAARGKSDWEIAKILGISHETVIQHLKQARERYGV

GKRAQLTVHALFDGTLTFVDVLRR-------------------

>Sphingobium_Sphingobium_SYK6~contig2_3317 # 3589384 # 3590091 # -1 # ID=2_3317;partial=00;start_type=GTG;rbs_motif=GGAGG;rbs_spacer=5-10bp;gc_cont=0.637

----------------------------------------------M-------------

---------------------------------------DDILK---------RIAGATT

LPLLWRQMTRYYHRHGFGGVSYYWVRT------TTNLPASVPLQYGFSKSEI---ELYLS

FDFQRLDIVPRAALAAGMPIRWSDIWKNSE-------LTGEEREFLAAMRTIDF----TD

GFSLPC-YGPNSRNAV-VGIGKMTEQTD----ISPSHLSLLHFAAQAAHLRICTLFA---

-------DEVVRDRQLSTREKEILDWVARGKSNNVIAEILAISPGTVDTYMRRIYEKLEV

SDRTSAAVKGVGLGLIAA-------------------------

>Sphingobium_Sphingobium_UT26S~contig2_447 # 479475 # 480152 # -1 # ID=2_447;partial=00;start_type=TTG;rbs_motif=AGGA;rbs_spacer=5-10bp;gc_cont=0.634

------------------------------------------------------------

------------------------------------------------------------

---MSDLMAEITKAMGFRHYALVHHVD-------LKPAVRSVHIVDYPQDWV---ERFQA

RRLYASDPIHRASHRTNVGFAWSAVQSIIS-------LTAADRSILAEAYEAGL----GD

GFTVPA-HIPGELNGS-CSFAMAAGEML-----DHRQLPFVQLVGSFAFEAARKISRIH-

-------APQSEWPSLTERQAECVALVARGKTDWEISQILGIGQETVIQHVKDARDRYGV

TKRTLLAIRALFDGQISFADVFGR-------------------

>Sphingobium_Sphingobium_UT26S~contig2_448 # 480235 # 480996 # -1 # ID=2_448;partial=00;start_type=ATG;rbs_motif=GGA/GAG/AGG;rbs_spacer=11-12bp;gc_cont=0.630

M---------------------------------------------ANRIKDCGR-----

-----------------------------------LDDLTRFAT---------DCQRAET

LGDLHGTIDAAVRDLGFRWFTLLHNID------LRRGDEQSLFLTTYPSAWL---EEVLE

ERHYIEDPIHAACARTPSGLAWDRVGDMLE-------LTPRQRGILDRARDHDL----AS

GYSLPI-RTPGEPEAI-FTAARSRDEPL-----SAEEVLTARLLGSVAYDRARELLGER-

-------AGPLACVPLSPRQVECIALVAQGKSDWEIGQILGLSRDTVHEYVESARRRYGV

RRRTQLVLRAVRDGHLNMEALL---------------------

>Sphingobium_Sphingobium_UT26S~contig2_2849 # 2984088 # 2984855 # 1 # ID=2_2849;partial=00;start_type=ATG;rbs_motif=GGA/GAG/AGG;rbs_spacer=5-10bp;gc_cont=0.626

----------------------------------------------MSM-----------

-----------------------------------HQEAMHFLQ---------CINQVRT

RDDMGSVMRVIAEQLGFQYFALTQHVD------VVAAGSGVIHIHNYPDRWA---DFYAA

NALGITDPVHRACHMTSWGFRWTQMPTLIP-------LTSGDHRHLARGRQAGI----GD

GFTIPS-NIGGQPPGS-CTFANEGDRPL-----REDRLLLAQLLGNYAFDAARRLWSMP-

-------RRAMTAPLITDRQRDCVQWAARGKSDWETSQILDISRETVTSHIKDACARYQV

NKRILLVGRTLGDGTLTISDIGGWSHPHFWE------------

>Sphingomonas_AAQG01~contig7_112 # 102651 # 103421 # -1 # ID=7_112;partial=00;start_type=TTG;rbs_motif=3Base/5BMM;rbs_spacer=13-15bp;gc_cont=0.607

----------------------------------------------MIGDAGQL------

-----------------------------------AALADDLLR---------ALRKVAS

REELAALMTCLASEIGFRHFALIHHDP------LAGERPDRVDIRDYPAAIV---DRLFD

QGQLRRDPVIRGCLFSDGAFLWSDLDKIIT-------FTRQDRHSLEFGAGHGL----NE

GITVPH-VLLGECIGS-CTFAGATDPQL-----ASRYLGFAQITGIFAFQAARRMLLAS-

-------PLIGPAPRLHPRPRDCVVLAGRGFSNKEIARELGLTPRTVDGYLTEARRLFDA

HDRAELVVSAVLAGEIGLDELKPRQTE----------------

>Sphingomonas_AAQG01~contig9_48 # 37907 # 38653 # -1 # ID=9_48;partial=00;start_type=ATG;rbs_motif=None;rbs_spacer=None;gc_cont=0.627

---------------------------------------------MMIG-----------

-----------------------------------LGQVQDFCA---------VAGAIKD

ATALSDLMAEITKAMGFRHYALVHHVD-------LKPAVRSVHIVDYPQDWV---ERFQA

RRLYASDPIHRASHRTNVGFAWSAVQSIIS-------LTAADRSILAEAYEAGL----GD

GFTVPA-HIPGELNGS-CSFAMAAGEML-----DHRQLPFVQLVGSFAFEAARKISRIH-

-------APQSECPSLTERQAECVALVARGKTDWEISQILGIGQETVIQHVKDARDRYGV

TKRTLLAIRALFEGQISFADVFGR-------------------

>Sphingomonas_AAQG01~contig9_49 # 38667 # 39428 # -1 # ID=9_49;partial=00;start_type=ATG;rbs_motif=GGA/GAG/AGG;rbs_spacer=11-12bp;gc_cont=0.636

M---------------------------------------------ANRIKDCGR-----

-----------------------------------LDDLTRFAT---------DCQRAET

LGDLHGTIDAAVRDLGFRWFTLLHNID------LRRGDEQSLFLTTYPSAWL---EEVLE

ERHYLEDPIHAACARTPSGLAWDRVGDVLE-------LTPRQRGILDRARDHDL----AS

GYSLPI-RTPGEPEAI-FTAARSRDEPL-----SAEEVLTARLLGSVAYDRARELLGER-

-------AGPLACVPLSPRQVECIALVAQGKSDWEIGQILGLSRDTVHEYVESARRRYGV

RRRTQLVLRAVRDGHLNMEALL---------------------

>Sphingomonas_AFGG01~contig50_9 # 9467 # 10186 # -1 # ID=50_9;partial=00;start_type=ATG;rbs_motif=GGxGG;rbs_spacer=5-10bp;gc_cont=0.671

----------------------------------------------MSGAA---------

-----------------------------------LAGAAAAVA---------AIEHSMD

AETLHATVRAYAAPFGYGRFVIYTAPPPGDG--------AVDRILWLEGDWFGNGHRVNA

ATYLARCPVNRHVLETDRPFFWTKNGT----------ADAETYRVVPHPKGPGI-----H

GLQVPI-FSHVGLAGA-VSFGGRTIDSA------IDTRLALTLVGTAAFHVAERLAGS--

-------VVAHAMPQLSAREREVIRWIASGRRQADVAALLGLSERTIENHLRRIRNRLGA

ASTAQAIHLLVRSGNLGA-------------------------

>Sphingomonas_AFMP01~contig51_16 # 11832 # 12620 # -1 # ID=51_16;partial=00;start_type=ATG;rbs_motif=GGA/GAG/AGG;rbs_spacer=5-10bp;gc_cont=0.619

----------------------------------------------MSPDRSAVRG----

--------------------------------RSWLALADAYIH---------SIRSAAT

ESDLRTLMEAVAHEIGCRHFALIHHDD------FRQERQDRVDLKDYPSAIT---ERLFG

QRRYRRDPVIRACVFADSAFLWSDLPRIIQ-------LDGQDRASLEFGLAEGL----NE

GITVPC-IRLGECMGS-CTFAGMRHPHN-----ASSYLGPAQMLGIFAFQAARRLLPAG-

------QSIPAARPRLHPRHRDCVVLAGRGLSNKEIARALALTPRTVDGYLTEARRLFDA

HDRTELVVSAVLAGEVGLHELRPRQPE----------------

>Sphingomonas_AGFU01~contig67_96 # 92437 # 93195 # 1 # ID=67_96;partial=00;start_type=GTG;rbs_motif=AGGAG/GGAGG;rbs_spacer=11-12bp;gc_cont=0.549

----------------------------------------------MGRRLDHAR-----

-----------------------------------LNDFDEVRK---------ACAKVVT

TAQLSSVIDAAIREFGFRWFALVHDGH------LQKQRPEQLMMTNYPASWV---DEVIS

DRLYMHDPVHIASARSAFGLCWERIADVIA-------PTRRQLSVLTRGRDHGL----VS

GFTIPF-RIPGERGAF-FTVARKRDRPF-----SNVEMMTAQLVAGVAFQRGRELVGGI-

-------EATIPSVSLSPRQIQCLELIAEGKTDWEIGTILGLSQNTIHEYIEEARHRYQV

KTRSQLVLAAARDGKISFNHMK---------------------

>Sphingomonas_AHHA01-1~contig2_96 # 113335 # 114156 # -1 # ID=2_96;partial=00;start_type=ATG;rbs_motif=AGxAGG/AGGxGG;rbs_spacer=5-10bp;gc_cont=0.669

----------------------------------------------MLISRVPHDDVGQT

----------------------FSNVFIAKTVPRAVPPAKDSSA---------VIETCRT

AAELRTWLLKFARGLGFYGARYIQLRRLCWGLGDADSAQPIRYLSTSSQ------ADRED

EQWIRADPSIGQIRNAFAPFAWSTRSGQD--------VTPQQRAWLDGERSRGI----DA

GLTIPV-QDSEGCPAY-LNLFGIDEAAV--RDLIDARAAELAFTAAQFHARAKALVPVA-

-------HWVGHGPKLSNREIECLRLAAFGQNVNESGQTLGISGRTVEFHLRNALDKLGA

PTKIRAVVLAFGTGIVAAQA-----------------------

>Sphingomonas_AIDW01~contig23_93 # 102796 # 103617 # -1 # ID=23_93;partial=00;start_type=ATG;rbs_motif=AGxAGG/AGGxGG;rbs_spacer=5-10bp;gc_cont=0.668

----------------------------------------------MLISRVPHDDVGQT

----------------------FSNVFIAKTVPRAVPPAKDSSA---------VIETCRT

AAELRTWLLKFARGLGFYGARYIQLGRLCWGLGDADSAQPIRYLSTSSQ------ADRED

EQWIRADPSIGQIRNAFAPFAWSTRSGQD--------VTPQQRAWLDGERSRGI----DA

GLTIPV-QDSEGCPAY-LNLFGIDEAAV--RDLIDARAAELAFTAAQFHARAKALVPVA-

-------HWVGHGPKLSNREIECLRLAAFGQNVNESGQTLGISGRTVEFHLRNALDKLGA

PTKIRAVVLAFGTGIVAAQA-----------------------

>Sphingomonas_ASTM01~contig10_193 # 206384 # 207133 # -1 # ID=10_193;partial=00;start_type=ATG;rbs_motif=GGA/GAG/AGG;rbs_spacer=5-10bp;gc_cont=0.577

----------------------------------------------MDR-----------

-----------------------------------VKDFDKFIY---------RMRDARD

LSVLDDVMCEVTSVFGFDQFALGHHVD------LVRPPRDAIRLTNYDPSWI---NESLE

QQFFADDPVHAVSAKLVRPFFWREMADHLA-------LTDHQQLILDRARSFRL----YD

GITAPV-HLPGEYEGS-CSFATDDFSKV-----HPMALALSHTAATFAFEGARRLMRQL-

-----DGKKAEPVPQFTNKEREILILVGRGKTDAEIGMVLGISKNTAHQHVETGRRAYGN

AQRTLMVIRAVFDGVINFADILR--------------------

>Sphingomonas_ASTM01~contig15_29 # 37431 # 38336 # 1 # ID=15_29;partial=00;start_type=ATG;rbs_motif=GGAG/GAGG;rbs_spacer=5-10bp;gc_cont=0.667

MHDRQDGALGAPQQAGYAPGRRRTSMAGQSFHRIRDCGRAST----MSGNPSAFDA----

-------------------------------NGMQMAAFSSLVV---------GLEASRS

MDEAMDVLHGAILELGFPRVVYGCVSIAHLP--SGTWVPAPLQVRGFPDRWD---RDWPR

HR--AHDPYAHTAFLKMESTNWTVVQNNAD------LLDPAQVDCISYINDLGL----NP

GLTVPL-FIPGHHYGF-ITAVGDGGLDG-WDAAADRAGPTLAMIANYFDNFAIRRFG---

-------GPPKESQSLSKRELECLTWSARGKTVEDIAVILDLSADTVRVYLKRVNQKLDA

VNRSHAVAKAMCLGMIDIS------------------------

>Sphingomonas_ASTM01~contig105_43 # 50967 # 51737 # -1 # ID=105_43;partial=00;start_type=ATG;rbs_motif=GGA/GAG/AGG;rbs_spacer=5-10bp;gc_cont=0.610

----------------------------------------------MPRSTMASE-----

-----------------------------------SQFAEDFLA---------AVRATAS

ESELCALMVAASRELGFRHYALIHHSL------PSDDSFGRIDMKDYPQAVE---KRLFQ

EGHFRRDPILRACLFADSAFLWSDLDRYID-------LDRRDKASLAFGLALGL----NQ

GITVPC-VLLGDCIGS-CTFAGTRHPDL-----AERRLGVVQVAGIFAFQAARRLSGTV-

-------HRRSPSPQLHPRPRDCVVLAGRGLSNKEIARALALSPRTVDGYLTEAREMFGA

HDRTELVVSAVLAGEVGLHELRC-QPE----------------

>Sphingomonas_ASTM01~contig106_37 # 39082 # 39912 # -1 # ID=106_37;partial=00;start_type=ATG;rbs_motif=None;rbs_spacer=None;gc_cont=0.706

----------------------------------------------MERADDE-------

-----------------------------------IPGGGSLAA-GAV-----AIRSPAD

IRPAAIHVRDVAARIANLRVAATDNIAARVPMRDEEGEVLATTVFAWDPDGA---QWWQD

VRFGLRAPVAEACRVESRPFWANRDGARDR-------DGTPILRQFDFAAHYGRLVENPA

GIVVPV-HLPFSRIGM-VSFSCRDNRRDDLSRELDEHFETLYLLGHLFIEGYARLETGD-

-------RWLPDAVTLTRLEVNCLRWVARGKTDDEIATIMGRARPTIRFHLQNAAIKLGA

ANRSQAVFRAGQLGYLSAGSAP------PALTVV--DR-----

>Sphingomonas_JFYY01.1~contig11_24 # 26858 # 27649 # 1 # ID=11_24;partial=00;start_type=ATG;rbs_motif=AGGAG;rbs_spacer=5-10bp;gc_cont=0.602

----------------------------------------------MYV-----------

-----------------------------------NARMRETLS---------RFNAASS

MEALLAALADAATKMGFPYVAMIQHGG------LPRLVERALVITNYPAEFV---RFYTE

SHAYVIDPVYEVSQLLDRPFSWDEIPGYVD-------LTGTQSALFEEARLHGL----VH

GVTVPL-HIPSESHAS-CTFARAEPIMA-----SPSLLTTLHIVAAFGFKAGLRLHHA--

-------SRGHDVPRLTRREAQCTALVAVGKSDWEISQILGLSETSVRYFVSHAKQRYGV

YKRSELVARALIDAQILRNDQGIIEAGPRRPRHRAKRRS---P

>Sphingomonas_JFYY01.1~contig16_93 # 87394 # 88149 # 1 # ID=16_93;partial=00;start_type=ATG;rbs_motif=GGA/GAG/AGG;rbs_spacer=5-10bp;gc_cont=0.574

----------------------------------------------MLM-----------

-----------------------------------SPRMIEFID---------GCREITD

EAGLREMLREMTKALGFNQYALLHHVD------LARPPAHAITLMGYEDGWM---EQILQ

NRYFQDDPILTASHRRLTGFSWREVPNIIR-------LNRRHRRILHQARAHGL----RD

GFTVPVPPLAGEYRGT-CSFASDRPVEI-----TSDLIGSAQLIATFCFEVARRITNQD-

------AVSAPRMPQLTQRQLDCIVLVGTGKTSWEIGKIIGLSEDTIDDHLSNAMRKCDV

SRRIQLVVRALFEGQASYWDMLRMS------------------

>Sphingomonas_JFYY01.1~contig27_42 # 37549 # 38301 # -1 # ID=27_42;partial=00;start_type=ATG;rbs_motif=AGxAG;rbs_spacer=5-10bp;gc_cont=0.595

----------------------------------------------MR------------

-----------------------------------LGLVELLGD---------GILKASS

IDHLHEALARATRDMDFDRFALSLEIG------WGADSSTSLLIHDYPASWA---DVYIG

FNLAATDPVRRAAERSVLGFGWRNILDLVP-------MTDMEKTTFETGRRHGL----VD

GFTVPR-HLPGDVIGS-CSFVTGLEKSI-----PHTMLVVAEMLGAMAIASARQLSGW--

-------PIRSAAPKLTDRQRDCVLWAARGKTDWEISRILDISHETVIQHLKDARERYET

HKRASLILYALYDGLISFADIFRWRVRR---------------

>Sphingomonas_JOOE01.1~contig1_414 # 425195 # 425941 # 1 # ID=1_414;partial=00;start_type=ATG;rbs_motif=GGA/GAG/AGG;rbs_spacer=5-10bp;gc_cont=0.657

----------------------------------------------MHR-----------

-----------------------------------LLLAQEFEH---------RTKDLKA

VADLEDVLGEACSAMGFRYFALMHHVD------TAEGPGPSLRLHNYPLSFS---SWFDE

NRFGRLDPVHRACHRTTKGFRWAHVPAMIR-------LGGTDFEILERARREGI----AN

GFTVPA-HVPGESRGS-CSFAVSEAEDV-----PDDWLPVAQLIGVCAFETARRLMLPD-

-------GPSGGPLRLTARQRDCVIWAARGKTDWEIGRILGLSKETVRQHLKQARERYGI

QKRSQLAVRALYDGVISFGEVLDS-------------------

>Sphingomonas_Sphingomonas_MM1~contig1_204 # 204979 # 205740 # 1 # ID=1_204;partial=00;start_type=ATG;rbs_motif=GGA/GAG/AGG;rbs_spacer=11-12bp;gc_cont=0.635

M---------------------------------------------ANRIRDCGR-----

-----------------------------------LDDLTRFAQ---------ECARVGN

LGDLQGTIDAAVRELGFRWFTLLHNVD------LRRGCGESLFLTTYPSAWL---EEVLA

ERHYVEDPIHAACARTPSGLAWDRVGDVLE-------LSSRQRSILKRARDHDL----AA

GYSLPI-RTPGEPEAI-FTVARPRDEPL-----DAAEILTARLLGSVAYDRARELLGEG-

-------IRTFVFVPLSPRQVECIALVAQGKSDWEIAQILGLSRDTVHEYVESARRRYGV

RRRTQLVLRAVRDGHLNMDALL---------------------

>Sphingomonas_Sphingomonas_MM1~contig1_205 # 205757 # 206500 # 1 # ID=1_205;partial=00;start_type=ATG;rbs_motif=None;rbs_spacer=None;gc_cont=0.610

----------------------------------------------MIG-----------

-----------------------------------LGQVQEFCA---------VAATIKD

SRALAGLMAEITREMGFRYFALVHHID-------LKPAVSAVHIVDYPPEWV---ERFQA

RRLYASDPIHRASHRTNIGFAWSAVQSLIT-------LSAADRSILAEAHDAGL----GD

GFTVPA-HIPGEVNGS-CSFAMGSGDVL-----DQRQLPLVQLIGSFAFEAARRLFRQT-

-------QAPCEGPSLTERQAECVALVARGKTDWEISQILGIGQETVIQHVKDARDRYGV

TKRTLLAIRALFDGQISFADVFGR-------------------

>Sphingomonas_Sphingomonas_wittichii~contig3_177 # 165609 # 166367 # -1 # ID=3_177;partial=00;start_type=ATG;rbs_motif=GGA/GAG/AGG;rbs_spacer=5-10bp;gc_cont=0.581

----------------------------------------------MVQAIDTAQ-----

-----------------------------------LRDFEEVHK---------ACEAAES

SAQLSEVIAGAIQQFGFRWFALVDDGD------LYKHRSGCMMLTNYPSSWV---DEVIS

ARLYRDDPVHAASIRSPTGLCWDRIPDVIA-------PTRAQLSVLERGRAHGL----TT

GYTVPF-RIPGERGAF-FSIARPKDRSF-----TYSEAISAQLLGGIAFEAGRALVKGR-

-------AVKAHGAPLTPRQIDCLRLIAAGKTEWEMGKILGLSPSTIHEYVEAARRRYGV

KTRSQLVLAAARDGYVSLNYLT---------------------

>Sphingomonas_Sphingomonas_wittichii~contig3_2024 # 2237514 # 2238350 # -1 # ID=3_2024;partial=00;start_type=ATG;rbs_motif=None;rbs_spacer=None;gc_cont=0.703

----------------------------------------------MERADDE-------

-----------------------------------IPGGGSLAA-GAV-----AIRSPAD

IRPAAIHVRDVAARIANLRVAATDNIAARVPMRDEEGEVLATTVFAWDPDGA---QWWQD

VRFGLRAPVAEACRVESRPFWANRDGARDR-------DGTPILRQFDFAAHYGRLVENPA

GIVVPV-HLPFSRIGM-VSFSCRDNRRDDLSRELDEHFETLYLLGHLFIEGYARLETGD-

-------RWLPDAVTLTRLEVNCLRWVARGKTDDEIATIMGRARPTIRFHLQNAAIKLGA

ANRSQAVFRAGQLGYLSAGSAPQA----PALTVV--DR-----

>Sphingomonas_Sphingomonas_wittichii~contig3_4410 # 4813199 # 4814104 # 1 # ID=3_4410;partial=00;start_type=ATG;rbs_motif=GGAG/GAGG;rbs_spacer=5-10bp;gc_cont=0.663

MHDRQDGALGAPQQAGYAPGRRAISMAGQSFHRIRDCGRAST----MSGNPSAFDA----

-------------------------------NGMQMAAFSSLVV---------GLEASRS

MDEAMDVLHGAILELGFPRVVYGCVSIAHLP--SGTWVPAPLQVRGFPDRWD---RDWPR

HR--AHDPYAHTAFLKMESTNWTVVQNNAD------LLDPAQVDCISYINDLGL----NP

GLTVPL-FIPGHHYGF-ITAVGDGGLDG-WDAAADRAGPTLAMIANYFDNFAIRRFG---

-------GPPKESQSLSKRELECLTWSARGKTVEDIAVILDLSADTVRVYLKRVNQKLDA

VNRSHAVAKAMCLGMIDIS------------------------

>PL|I2BZW4|PsoR|Pseudomonas_fluorescens_A506

----------------------------------------------M-------------

-----------------------------------LAKLTAFNN---------RLIPGRS

LDEQMDNTFILAQQLGFDALVYDYSPVPIDL-NGALITPLLLQLRNTPADWH---ALWCS

EGFYQIDPVQHLALSSVSPFVWSYEAKTETALQK--IIDPCHAPVSSYLHERQL----TC

GVSVPI-HLPKGGFAS-LTGLRTGKART-VLKDAQHTLADFSLITHALQEAAYPLFSKE-

-------VRAYPHIRLTKRERECLKWAAEGLTAAEIATQLSRSLAVVTLHLASAMHKLGA

KNRVQAVVRATHYRLLED-------------------------

>PL|M4MVH9|NesR|Sinorhizobium_meliloti_Sm2011

----------------------------------------------M-------------

-----------------------------------FDELGTIRN---------QFTAHDT

LDGRIDQVFEAMKSIGFEALIYDYTPVPRDL-DGTIMVPSLLKLRNISEDMH---DYWFD

RGYFRIDPVQQVALRTSTPFFWNYDPDADTLIRR--FMSDDTAPVARYLSERDM----ST

GVTVPV-HMPRGDYAT-VTGVRFGGNRA-FEGHALRYIADFNLLAHVFHEAAYSLFDAQ-

-------AFNAGTARLTERERECLRYSAEGHSAKEISRIIHRSVPTVVMHLNAAAKKLGA

KNRTQAVVRATHYRLLEERPSYNL-------------------

>PL|H1XM29|XagR|Xanthomonas_axonopodis

----------------------------------------------M-------------

-----------------------------------FDILASLGR---------DLQALRT

LDSCLDRVFGDVCALGFQSLVYDYAPVPLSL-EGALITPTVFMQRNAPGDMQ---HVWCE

HGYYQHDPVQQRATRHTMPFVWSYRTDGHCPGVE--YVGGQHRQVTRYLCDSGM----GT

GVTVPL-HLPGGAFAT-FSGAIDAAAAQ-APRLAEAQLLPFLLLAHAFQSRAQELLDPQ-

-------ERRCHHIALTRRERECLQYSAKGLTSKRIAAALNRSTATVNLHLNSAARKLGA

RNRVEAVVRGMHYRLLEP-------------------------

>PL|Q3BQU7|XccR|Xanthomonas_campestris

----------------------------------------------M-------------

-----------------------------------FDILASLGC---------DLQALQT

LDSCLDRVFRDVCALGFQSLVYDYAPVPLSL-EGALITPTVFMQRNAPGDMQ---HVWCE

HGYYQHDPVQQRATRRTTPFVWSYRTDGHCAGVE--YVGGQHRQVTRYLCDSGM----GT

GVTVPL-HLPGGAFAT-FSGAIDAAAAQ-APRLAEAQVLPFLLLAHAFQARAQELLEPQ-

-------ERRCHHIALTRRERECLQYSAKGLTSKRIAAALNRSTATVNLHLNSAARKLGA

RNRVEAVVRGMHYRLLEP-------------------------

>PL|Q2P6A5|OryR|Xanthomonas_oryzae

----------------------------------------------M-------------

-----------------------------------FEILASLGR---------DLQASQT

VNSCLDRVFRDVCALGFQSLVYDYAPVPLSM-EGALITPTVFMQRNAPGDMQ---HVWCE

HGYYQHDPVQQRATRRNTPFVWSYRTDGDCAGVE--YVGGQHRQVTRYLCDSGM----GT

GVTVPL-HLPGGAFAT-FSAAIDAVAAE-ALRLAESQLLPFLLLAHAFQARAQELLDPQ-

-------ERRCHHIPLTRRERECLQYSAKGLTSKRIAAALNRSTATVNLHLNSAARKLGA

RNRVEAVVRGMHYRLLEP-------------------------

>sp|G2L851|QscR|Pseudomonas_aeruginosa_M18

----------------------------------------------M-------------

-----------------------------------HDEREGYLE---------ILSRITT

EEEFFSLVLEICGNYGFEFFSFGARAP------FPLTAPKYHFLSNYPGEWK---SRYIS

EDYTSIDPIVRHGLLEYTPLIWNGE------------DFQENRFFWEEALHHGI----RH

GWSIPV-RGKYGLISM-LSLVRSSESIA--ATEILEKESFLLWITSMLQATFGDLLAPR-

-------IVPESNVRLTARETEMLKWTAVGKTYGEIGLILSIDQRTVKFHIVNAMRKLNS

SNKAEATMKAYAIGLLN--------------------------

>sp|Q7NQP7|CVIR|Chromobacterium_violaceum

----------------------------------------------MVTSKPINARP--L

------------------------------PAGLTASQQWTLLE------WIHMAGHIET

EGELKAFLDNILSQAPSDRIILVLGRLNNQ---NQIQRMEKVLNVSYPSDWL---NQYSQ

ENFAQHDPIMRIHLGQ-GPVIWEERFSRA--------KGSEEKRFIAEASSNGM----GS

GITFSA-ASDRNNVGSILSIGGKEPGRN------AALVAMLNCLTPHLHQAAVRIANLP-

-------PASPSNMPLSQREYDIFHWMSRGKTNWEIATILNISERTVKFHVANVIRKLNA

NNRTHAIVLGMHLAMTPRELVNG--------------------

>sp|P0A3J6|AHYR|Aeromonas_salmonicida

----------------------------------------------MK------------

-----------------------------------QDQLLEYLE---------HFTSVTD

GDRLAELIGRFTLGMGYDYYRFALIIP------MSMQRPKVVLFNQCPDSWV---QAYTA

NHMLACDPIIQLARKQTLPIYWNRLDERAR------FLQEGSLDVMGLAAEFGL----RN

GISFPL-HGAAGENGI-LSFITAERASS---DLLLESSPILSWMSNYIFEAAIRIVRVS-

------LREDDPQEALTDRETECLFWASEGKTSGEIACILGITERTVNYHLNQVTRKTGS

MNRYQAIAKGVSSGILLPNLEQVVVTNFPKLMQ----------

>sp|P0A3J5|AHYR|Aeromonas_hydrophila

----------------------------------------------MK------------

-----------------------------------QDQLLEYLE---------HFTSVTD

GDRLAELIGRFTLGMGYDYYRFALIIP------MSMQRPKVVLFNQCPDSWV---QAYTA

NHMLACDPIIQLARKQTLPIYWNRLDERAR------FLQEGSLDVMGLAAEFGL----RN

GISFPL-HGAAGENGI-LSFITAERASS---DLLLESSPILSWMSNYIFEAAIRIVRVS-

------LREDDPQEALTDRETECLFWASEGKTSGEIACILGITERTVNYHLNQVTRKTGS

MNRYQAIAKGVSSGILLPNLEQVVVTNFPKLMQ----------

>sp|Q89VI3|BJAR1|Bradyrhizobium_diazoefficiens

----------------------------------------------MSAVD---------

----------------------------------YGREALDFIE---------GLGVYRK

VPDAMNALEAAFGRFGFETIIVTGLPN------PDQRFAQMVLAKRWPAGWF---NLYTQ

NNYDRFDPVVRLCRQSVNPFEWSEAPYDAE-------LEPSAAEVMNRAGDFRM----SR

GFIVPI-HGLTGYEAA-VSLGGVHLDLN------PRSKPALHLMAMYGFDHIRRLLE---

-------PTPYPSTRLTPREREVISWASQGKSAWEIGEILHITQRTAEEHLATAARKLGA

VNRTHAVALAIRHKIINP-------------------------

>sp|Q46751|CARR|Pectobacterium_carotovorum_subsp._carotovorum

----------------------------------------------M-------------

------------------------------------------------------------

DHEIHSFIKRKLKGVGDVWFSYFMM--------SKNSTSQPYIISNYPEAWM---KEYIK

KEMFLSDPIIVASLARITPFSWDDNDIVT--------LRAKNQDVFISSVQHDI----SS

GYTFVL-HDHDNNVAT-LSIANHLEDAN-FEKCMKNHENDLQMLLVNVHEKVMAYQRAI-

-NDQDNPPDNSRNALLSPRETEVLFLVSSGRTYKEVSRILGISEVTVKFHINNSVRKLDV

INSRHAITKALELNLFHSPCEPVVMKHMDAR------------

>sp|Q46967|ECHR|Dickeya_chrysanthemi

----------------------------------------------MSIS----------

-----------------------------------------------------FSNFDFI

NSTIQNYLNRKLKSYGDLKYAYLIM--------NKKKPTDVVIISNYPSEWV---EIYRS

NNYQHIDPVILTAINKISPFSWDDDLVIS--------SKLKFSRIFNLSKEYDI----VN

GYTFVL-HDPGNNLAT-LSFMFEENRSGELEEIVQNNKEKLQMLLISAHEKLTSLYREM-

-SKNKNNSKSQEPNIFSQRENEILYWASMGKTYQEIALILGITTSTVKFHIGNVVKKLGV

LNAKHAIRLGVEMNLIKPVEPVKARS-----------------

>sp|P54293|ESAR|Pantoea_stewartii_subsp._stewartii

----------------------------------------------MFSF----------

-----------------------------------------------------FLENQTI

TDTLQTYIQRKLSPLGSPDYAYTVV--------SKKNPSNVLIISSYPDEWI---RLYRA

NNFQLTDPVILTAFKRTSPFAWDENITLM--------SDLRFTKIFSLSKQYNI----VN

GFTYVL-HDHMNNLAL-LSVIIKGNDQTALEQRLAAEQGTMQMLLIDFNEQMYRLAGTE-

GERAPALNQSADKTIFSSRENEVLYWASMGKTYAEIAAITGISVSTVKFHIKNVVVKLGV

SNARQAIRLGVELDLIRPAASAAR-------------------

>sp|Q47188|EXPR|Dickeya_dadantii

----------------------------------------------MSIS----------

-----------------------------------------------------FSNVDFI

NSTIQSYLNRKLKSYGDPKYAYLIM--------NKKKPTDVVIISNYPTEWV---DIYRN

NNYQHIDPVILTAINKISPFSWDDDLVIS--------SKLKFSRIFNLSKDYDI----VN

GYTFVL-HDPGNNLAA-LSFMIEEHRSEELEEIIQNNKDKLQMLLISAHEKLTSLYREM-

-SRNRNNSKSQEADLFSQRENEILHWASMGKTYQEIALILGITTSTVKFHIGNVVKKLGV

LNAKHAIRLGVEMNLIKPVGPAKARS-----------------

>sp|Q47189|EXPR|Pectobacterium_sp.

----------------------------------------------MSQL----------

-----------------------------------------------------FYNNETI

SRIIKSQFDMALSHYGDIKYAYMVL--------NKKKPTEILIISNHHDEWR---EIYQA

NNYQHIDPVVIAALNKITPFPWDEDLLVS--------TQLKMSKIFNLSREHNI----TN

GYTFVL-HDHSNNLVM-LSIMIDESNVSNIDDVIESNKDKLQMTLMTIHAETISLYREM-

-IRNKEDERSNDKDIFSQRENEILYWASMGKTYQEIALILDIKTGTVKFHIGNVVKKLGV

LNAKHAIRLGIELQLIRPVQS----------------------

>sp|P25084|LASR|Pseudomonas_aeruginosa_

----------------------------------------------MALVDGFL------

-----------------------------------------------------ELERSSG

KLEWSAILQKMASDLGFSKILFGLLPK------DSQDYENAFIVGNYPAAWR---EHYDR

AGYARVDPTVSHCTQSVLPIFWEPSIY----------QTRKQHEFFEEASAAGL----VY

GLTMPL-HGARGELGA-LSLSVEAENRAEANRFMESVLPTLWMLKDYALQSGAGLAFE--

-------HPVSKPVVLTSREKEVLQWCAIGKTSWEISVICNCSEANVNFHMGNIRRKFGV

TSRRVAAIMAVNLGLITL-------------------------

>sp|P12746|LUXR|Aliivibrio_fischeri

----------------------------------------------MKNIN---------

-----------------------------------ADDTYRIIN---------KIKACRS

NNDINQCLSDMTKMVHCEYYLLAIIYP------HSMVKSDISILDNYPKKWR---QYYDD

ANLIKYDPIVDYSNSNHSPINWNIFENNA--------VNKKSPNVIKEAKTSGL----IT

GFSFPI-HTANNGFGM-LSFAHSEKDNY-IDSLFLHACMNIPLIVPSLVDNYRKINI---

-------ANNKSNNDLTKREKECLAWACEGKSSWDISKILGCSERTVTFHLTNAQMKLNT

TNRCQSISKAILTGAIDCPYFKN--------------------

>sp|P35327|LUXS|Vibrio_fischeri_

----------------------------------------------MNIKN---------

-----------------------------------INANEKIID---------KIKTCNN

NKDINQCLSEIAKIIHCEYYLFAIIYP------HSIIKPDVSIIDNYPEKWR---KYYDD

AGLLEYDPVVDYSKSHHSPINWNVFEKKT--------IKKESPNVIKEAQESGL----IT

GFSFPI-HTASNGFGM-LSFAHSDKDIY-TDSLFLHASTNVPLMLPSLVDNYQKINT---

-------TRKKSDSILTKREKECLAWASEGKSTWDISKILGCSERTVTFHLTNTQMKLNT

TNRCQSISKAILTGAINCPYLKN--------------------

>sp|Q51786|PHZR|Pseudomonas_fluorescens

M---------------FK----------------------------MELGQ---------

----------------------------------LLGWDAYFYS---------IFAQAMN

MEEFTVVALRALRELRFDFFAYGMCSV------TPFMRPKTYMYGNYPEHWL---QRYQA

ANYALIDPTVKHSKVSSAPILWSNE------------LFRNCPDLWSEANDSSL----CH

GLAQPS-FNTQGRVGV-LSLARKDNAIS--LQEFEALKPVTKAFAAAALEKISALETDV-

-------RAFNTDVEFSERECDVLRWTADGKTSEEIGVIMGVCTDTVNYHHRNIQRKIGA

SNRVQAVSYAVALGYI---------------------------

>sp|P54303|PHZR|Pseudomonas_chlororaphis

----------------------------------------------MELGQ---------

----------------------------------QLGWDAYFYS---------IFARTMD

MQEFTAVALRALRELRFDFFRYGMCSV------TPFMRPRTYMYGNYPEDWV---QRYQA

ANYAVIDPTVKHSKVSSSPILASNE------------LFRGCPDLWSEANDSNL----RH

GLAQPS-FNTQGRVGV-LSLARKDNPIS--LQEFEALKVVTKAFAAAVHEKISELESDV-

-------RVFNTDVEFSGRECDVLRWTADGKTSEEIGVIMGVCTDTVNYHHRNIQRKIGA

SNRVQASRYAVAMGYI---------------------------

>sp|O54452|RAIR|Rhizobium_etli

----------------------------------------------MSPSH---------

-----------------------------------AEQFSFFLLSGPDLRIADIAGSGND

AGRSRPHLCDIAYGSPCD--------------LAGATDSNPLLMLTYPPEWV---KQYRD

RDYFSIDPVVRLGRRGFLPVEWSASGW----------DSGRAYGFFKEAMAFGV---GRQ

GVTLPV-RGPQGERSL-FTVTSNHPDAY-WRQFRMDSMRDLQFLAHHLHDRAMVLSGM--

-------RKVADLPRLSRRELQCLEMTANGLLAKQICARLSISVSAVQLYLASARRKLTV

ATTSEQLLGPRRSN-----------------------------

>sp|Q03316|RHIR|Rhizobium_leguminosarum_bv._viciae

----------------------------------------------MKEESSAV------

-----------------------------------SNLVFDFLS---------ESASAKS

KDDVLLLFGKISQYFGFSYFAISGIPS------PIERIDSYFVLGNWSVGWF---DRYRE

NNYVHADPIVHLSKTCDHAFVWSEALRDQK-------LDRQSRRVMDEAREFKL----ID

GFSVPL-HTAAGFQSI-VSFGAEKVELS------TCDRSALYLMAAYAHSLLRAQIGND-

-----ASRKIQALPMITTREREIIHWCAAGKTAIEIATILGRSHRTIQNVILNIQRKLNV

VNTPQMIAESFRLRIIR--------------------------

>sp|P54292|RHLR|Pseudomonas_aeruginosa_

----------------------------------------------MRNDGG--------

----------------------------------FLLWWDGLRS---------EMQPIHD

SQGVFAVLEKEVRRLGFDYYAYGVRHT------IPFTRPKTEVHGTYPKAWL---ERYQM

QNYGAVDPAILNGLRSSEMVVWSDS------------LFDQSRMLWNEARDWGL----CV

GATLPI-RAPNNLLSV-LSVARDQQNIS--SFEREEIRLRLRCMIELLTQKLTDLEHP--

-------MLMSNPVCLSHREREILQWTADGKSSGEIAIILSISESTVNFHHKNIQKKFDA

PNKTLAAAYAAALGLI---------------------------

>sp|P07026|SDIA|Escherichia_coli_

----------------------------------------------MQDKD---------

----------------------------------FFSWRRTMLL---------RFQRMET

AEEVYHEIELQAQQLEYDYYSLCVRHP------VPFTRPKVAFYTNYPEAWV---SYYQA

KNFLAIDPVLNPENFSQGHLMWNDD------------LFSEAQPLWEAARAHGL----RR

GVTQYL-MLPNRALGF-LSFSRCSAREI--PILSDELQLKMQLLVRESLMALMRLNDE--

-------IVMTPEMNFSKREKEILRWTAEGKTSAEIAMILSISENTVNFHQKNMQKKINA

PNKTQVACYAAATGLI---------------------------

>sp|P58590|SOLR|Ralstonia_solanacearum_

----------------------------------------------M-------------

-----------------------------------GPGFQDAYH---------AFHTAQD

ERQLFRQIASVVRQLGFDYCCYGIRVP------LPVSKPAVAIFDTYPAGWM---EHYQA

SGFLEIDPTVRTGASSSDLIIWPVS------------IRDEAARLWSDARDFGL----NI

GVARSS-WTAHGAFGL-LTLARRADPLT--AAELEQLSATTNWLANLAHALMSPFLMPK-

-------LVPESSAALTAREREVLCWTGEGKTAYEIGQILRISERTVNFHVNNVLLKLAA

TNKVQAVVKAIAIGLI---------------------------

>sp|O30919|SOLR|Ralstonia_solanacearum

----------------------------------------------M-------------

-----------------------------------EPDFQDAYH---------AFRTAED

EHQLFREIAAIARQLGFDYCCYGARMP------LPVSKPAVAIFDTYPAGWM---QHYQA

SGFLDIDPTVRAGASSSDLIVWPVS------------IRDDAARLWSDARDAGL----NI

GVARSS-WTAHGAFGL-LTLARHADPLT--AAELGQLSIATHWLANLAHTLMSPFLVPQ-

-------LVPESNAVLTTREREVLCWTGEGKTAYEIGQILRISERTVNFHVNNVLLKLAA

TNKVQAVVKAIATGLI---------------------------

>sp|P54294|TRAR|Agrobacterium_tumefaciens

----------------------------------------------M-------------

-----------------------------------QHWLDKLTD---------LAAIQGD

ECILKDGLADLAEHFGFTGYAY-----------LHIQHKHTIAVTNYHRDWR---SAYFE

NNFDKLDPVVKRAKSRKHVFAWSGEQERSR-------LSKEERAFYAHAADFGI----RS

GITIPI-KTANGSMSM-FTLASERPAID---LDREIDAAAAAGAVGQLHARISFLQTT--

-------PTVEDAAWLDPKEATYLRWIAVGMTMEEVADVEGVKYNSVRVKLREAMKRFDV

RSKAHLTALAIRRKLI---------------------------

>sp|P33909|TRAR|Agrobacterium_vitis

----------------------------------------------M-------------

-----------------------------------QHWLDKLTD---------LTAIEGD

GCILKTGLADVADHFGFTGYAY-----------LHIQHKHIIAVTNYHHDWR---SLYFD

KKFDALDPVVKRARSRKQVFAWSGEQERPK-------LSEEERAFYAQAADFGI----RS

GITIPI-RTANGSMSM-FTLASERTAIP---LDREIDAVAAAAAVGQLHARISFLRIT--

-------PTAEDAAWLDPKEATYLRWIAVGKTMEEIADVEEVKYNSVRVKLREAMKRFDV

RSKAHLTALAIKRKLI---------------------------

>sp|P33905|TRAR|Rhizobium_radiobacter

----------------------------------------------M-------------

-----------------------------------QHWLDKLTD---------LAAIEGD

ECILKTGLADIADHFGFTGYAY-----------LHIQHRHITAVTNYHRQWQ---STYFD

KKFEALDPVVKRARSRKHIFTWSGEHERPT-------LSKDERAFYDHASDFGI----RS

GITIPI-KTANGFMSM-FTMASDKPVID---LDREIDAVAAAATIGQIHARISFLRTT--

-------PTAEDAAWLDPKEATYLRWIAVGKTMEEIADVEGVKYNSVRVKLREAMKRFDV

RSKAHLTALAIRRKLI---------------------------

>sp|P55407|TRAR|Rhizobium_sp._

----------------------------------------------MSV-----------

-----------------------------------NGNLRSLID---------MLEAAQD

GHMIKIALRSFAHSCGYDRFAY-----------LQKDGTQVRTFHSYPGPWE---SIYLG

SDYFNIDPVLAEAKRRRDVFFWTADAWPAR-------GSSPLRRFRDEAISHGI----RC

GVTIPV-EGSYGSAMM-LTFASPERKVD--ISGVLDPKKAVQLLMMVHYQ--LKIIAAK-

-------TVLNPKQMLSPREMLCLVWASKGKTASVTANLTGINARTVQHYLDKARAKLDA

ESVPQLVAIAKDRGLV---------------------------

>sp|P74946|VANR|Vibrio_anguillarum

----------------------------------------------MYK-----------

--------------------------------------ILRLIQ---------ENQQITS

HDDLENVLNGLNNLIGHEFFLFGLSFQ------PTLKTSETLVTDNYPNSWR---QQYDE

SGFMHIDPIVKYSITNFLPIRWDDAKR----------VNNDGRVIFEEARCNGL----KA

GFSIPI-HGLRGEFGM-ISFATSDTKSY---DLNQQSIHTSQLIVPLLAHNIGNITRYH-

-------KDAKPRAVLTAREVQCLAWAAEGKSAWEIATIINTSERTVKFHFSNACKKLGA

TNRYQAITKAILGGYINPYL-----------------------

>sp|Q2YJ50|VJBR|Brucella_abortus_

----------------------------------------------MSLD----------

-----------------------------------LVHFPNYKK---------TFFGSSF

QSDTLALLTRIRDEIGCRYVTHTYRGRVGDC--TKVNSADLTVLMTLPATWV---ARYSS

KNYFAIDPVFQ----EDAPYYRNDTSAIARDLKEDADICPAVAELLHDAEKHGL---GNL

FIAVSA-RNPKGVAGC-TVFTFEVEDED-RTQFLARMRPRLLSLAGIIHGTVCGCK----

-------DANSVASLLTPREVDCLRWAANGKTDGEIAEILSIARWTVVTYLQNAKIKLNC

SNRTSAVATALSLGIIDMPEVQHLV------------------

>sp|Q8YAY5|VJBR|Brucella_melitensis_biotype_1

----------------------------------------------MSLD----------

-----------------------------------LVHFPNYKK---------TFFGSSF

QSDTLALLTRIRDEIGCRYVTHTYRGRVGDC--TKVNSADLTVLMTLPATWV---ARYSS

KNYFAIDPVFQ----EDAPYYRNDTSAIARDLKEDADICPAVAELLHDAEKHGL---GNL

FIAVSA-RNPKGVAGC-TVFTFEVEDED-RTQFLARMRPRLLSLAGIIHGTVCGCK----

-------DANSVASLLTPREVDCLRWAANGKTDGEIAEILSIARWTVVTYLQNAKIKLNC

SNRTSAVATALSLGIIDMPEVQHLV------------------

>sp|Q579W9|VJBR|Brucella_abortus_biovar_1

----------------------------------------------MSLD----------

-----------------------------------LVHFPNYKK---------TFFGSSF

QSDTLALLTRIRDEIGCRYVTHTYRGRVGDC--TKVNSADLTVLMTLPATWV---ARYSS

KNYFAIDPVFQ----EDAPYYRNDTSAIARDLKEDADICPAVAELLHDAEKHGL---GNL

FIAVSA-RNPKGVAGC-TVFTFEVEDED-RTQFLARMRPRLLSLAGIIHGTVCGCK----

-------DANSVASLLTPREVDCLRWAANGKTDGEIAEILSIARWTVVTYLQNAKIKLNC

SNRTSAVATALSLGIIDMPEVQHLV------------------

>sp|A5VTN1|VJBR|Brucella_ovis

----------------------------------------------MSLD----------

-----------------------------------LVHFPNYKK---------TFFGSSF

QSDTLALLTRIRDEIGCRYVTHTYRGRVGDC--TKVNSADLTVLITLPATWV---ARYSS

KNYFAIDPVFQ----EDAPYYRNDTSAIARDLKEDADICPAVAELLHDAEKHGL---GNL

FIAVSA-RNPKGVAGC-TVFTFEVEDED-RTQFLARMRPRLLSLAGIIHGTVCGCK----

-------DANSVASLLTPREVDCLRWAANGKTDGEIAEILSIARWTVVTYLQNAKIKLNC

SNRTSAVATALSLGIIDMPEVQHLV------------------

>sp|Q8FXF9|VJBR|Brucella_suis_biovar_1

----------------------------------------------MSLD----------

-----------------------------------LVHFPNYKK---------TFFGSSF

QSDTLALLTRIRDEIGCRYVTHTYRGRVGDC--TKVNSADLTVLMTLPATWV---ARYSS

KNYFAIDPVFQ----EDAPYYRNDTSAIARDLKEDADICPAVAELLHDAEKHGL---GNL

FIAVSA-RNPKGVAGC-TVFTFEVEDED-RTQFLARMRPRLLSLAGIIHGTVCGCK----

-------DANSVASLLTPREVDCLRWAANGKTDGEIAEILSIARWTVVTYLQNAKIKLNC

SNRTSAVATALSLGIIDMPEVQHLV------------------

>sp|P55629|Y4QH|Rhizobium_sp.

MPDLHRLALSALQ---YRP-------VGAQSGNASSAAASCTGD-YMAGDSSLERDCGRL

ADGCSFPQVRGSATGVSDQSLGGSIHFSNAGRDEYVVELGRLLD---------LTDGVAQ

PKKLFDLLSAFAFKFGCKWLAYGPLTSDHKA--LNRVKCDSEEILNYPDGWR---ERCLE

MGYETIAPVIKESRMGAGPIRWSDMYSDAS-------TTEYERRMFDEAAMFGL----RS

GITVPL-RGPRGSCAI-MSFARHCEREF-----HDRTIAYLQLAATHFHLRVAKIANL--

-------NAVQKIPALSLREKECVLWVARGKSSWDIGVIMRISENTVNFHIKNVMRKLGT

SSRTVAAIKAISLGIIEL-------------------------

>sp|P54295|YENR|Yersinia_enterocolitica

----------------------------------------------MIID----------

-----------------------------------------------------YFDNESI

NEDIKNYIQRRIKTYGDLCYSYLVM--------NKKTPLHPTIISNYPLDWV---KKYKK

NSYHLIDPVILTAKDKVAPFAWDDNSVIN--------KKSTDSAVFKLAREYNI----VN

GYTFVL-HDNSNNMAT-LNISNGSDDSISFDERIEINKEKIQMLLIITHEKMLGLYQSN-

SDKNENRNTQIERDIFSPRENEILYWASVGKTYAEISIILGIKRSTVKFHIGNVVRKLGV

LNAKHAIRLGIELKLIKPI------------------------

>sp|O87971|YUKR|Yersinia_ruckeri

------------------------------------------------------------

------------------------------------------------------------

-------IDRKLERYDSPRYTYMVI--------DKKNPVDVFIVTSYPDEWA---DIYTS

QNYQHIDPIVLTAFKRISPFAWDENITIL--------SDLKSSKIFALSKKYNI----VN

GFTFVL-HDHMNNLAM-LSLIMDNNADKGLNSRIESDKDRLQMNLIKIHEKMLMLEQNKL

GVSNGKNTDTSGKGILSPRENEVLHWASMGKTYPEIALIAGITTRTVKHHMGNVVKKLGV

INARQAIRLGVELELIKPVLV----------------------
