# Supplementary material for: Whole genome sequencing and analysis reveal insights into the genetic structure, diversity and evolutionary relatedness of luxI and luxR homologs in bacteria belonging to the Sphingomonadaceae family
Source: Front Cell Infect Microbiol. 2015 Jan 8;4:188. doi: 10.3389/fcimb.2014.00188 (PMC4288048; doi:10.3389/fcimb.2014.00188)
Supplement: Supplementary file 6 [file Image1.PDF]

Supplemental Figure 1.

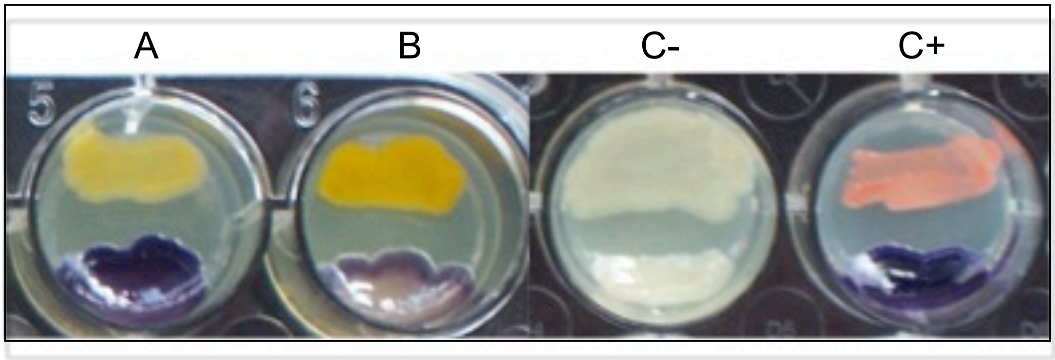

AHL detection bioassay with CviR-dependent whole cell sensor CV026.

Test strains top of each well; *Sphingomonas paucimobilis* EPA505, A; and *Sphingobium herbicidovorans* NBRC16415, B. Controls strains top of each well: *E.coli*, C-; *Methylobacterium* sp. GXF4, C+. CV026 biosensor strain on bottom half of each well. Violacein pigmentation development indicates test strain or controls produce AHL QS signals.
